# Work‐Related Stress Among Neglected Work Forces in Tertiary Hospitals, Eastern Ethiopia: A Cross‐Sectional Study

**DOI:** 10.1002/hsr2.73374

**Published:** 2026-10-10

**Authors:** Sina Temesgen Tolera, Dawit Kassaye Getaneh, Ararso Tafesse, Fethia Mohammed, Roba Argaw Tesema

**Affiliations:** ^1^ College of Health and Medical Sciences Haramaya University Harar City Ethiopia; ^2^ Ambo University College of Medicine and Public Health Ambo Ethiopia

**Keywords:** associated factors, cleaners, eastern Ethiopia, tertiary public hospitals, work‐related stress

## Abstract

**Background:**

Hospital cleaners are an often‐neglected workforce routinely exposed to hazardous working conditions that increase their risk of work‐related stress (WRS). However, evidence on WRS among hospital cleaners in low‐ and middle‐income countries, including Ethiopia is limited. This study aimed to determine the prevalence of WRS and identify associated factors among hospital cleaners working in eight tertiary hospitals in eastern Ethiopia.

**Methods:**

A hospital‐based cross‐sectional study was conducted among 741 cleaners from May to August 2023. Work‐related stress (WRS) was assessed using modified items from the Copenhagen Psychosocial Questionnaire II (COPSOQ‐II) and the National Health Survey. Severe and low WRS were classified using the mean cut‐off score derived from the summed components of physical indicators of WRS. Data quality was ensured through training, pretesting, expert evaluation of the assessment tools, and internal consistency testing using Cronbach's alpha. Data were entered into EpiData version 3.1 and analyzed using Stata 17MP. Descriptive statistics summarized participant characteristics. Binary and multivariable logistic regression analyses identified factors associated with WRS. Variables with *p* < 0.20 were included in the multivariable model, and AORs with 95% CIs were reported.

**Results:**

Of 741 hospital cleaners, 679 participated (response rate: 91.63%). The prevalence of severe WRS was 29.16% (95% CI: 25.77%–32.74%). Multivariable analysis identified significant factors. Workload (AOR = 10.23, 95% CI: 6.16–16.98; *p* < 0.001), smoking (AOR = 6.64, 95% CI: 3.53–12.51; *p* < 0.001), khat chewing (AOR = 4.07, 95% CI: 2.34–7.09; *p* < 0.001), job dissatisfaction (AOR = 2.67, 95% CI: 1.24–5.76; *p* = 0.012), less‐than‐monthly supervision (AOR = 1.63, 95% CI: 1.01–3.06; *p* = 0.030), lack of social recognition (AOR = 3.11, 95% CI: 1.63–5.92; *p* < 0.001), sleeping difficulties (AOR = 2.18, 95% CI: 1.27–3.71; *p* = 0.004), and inadequate OHS services (AOR = 1.64, 95% CI: 1.16–2.83; *p* = 0.043) increased WRS. Experience > 6.72 years reduced stress (AOR = 0.44, 95% CI: 0.26–0.75; *p* = 0.003).

**Conclusion:**

The study concludes that almost one‐third of cleaners in tertiary hospitals had severe WRS. Higher levels of stress were substantially associated with smoking, chewing khat, job dissatisfaction, infrequent supervision, lack of social recognition, trouble sleeping, and inadequate workplace health and safety services, with workload being the most significant predictor. Conversely, higher levels of education and more work experience were protective factors. These findings highlight the necessity of comprehensive workplace interventions that reduce excessive workloads, support occupational health and safety services, improve supervision and recognition, promote healthy lives, and support cleaners' professional growth.

## 1 Introduction

Work‐related stress (WRS) has been widely recognized as a major occupational health challenge across healthcare systems globally. Evidence indicates that healthcare workers, including non‐clinical staff such as hospital cleaners, are exposed to high psychosocial and physical demands that predispose them to stress, burnout, and related mental health problems [1]. Hospital cleaners worldwide deal with a demanding and varied job due to several potentially hazardous situations, including the possibility of WRS [2]. Particularly in hospitals and other healthcare facilities, employees are under a great deal of stress at work these days. It is thought of as an examination of the variations in stress outcomes among workers [3]. The mental pressure or distress brought on by organizational and personal pressures at work is known as job stress [4]. In the psychological sciences, WRS is a feeling of mental strain and tension; and excessive stress can cause major harm to people, as well as bodily, psychological, and social problems [5].

Recent systematic reviews indicate substantially higher prevalence estimates, varying by occupation and country, with approximately 39% of the global public health workforce experiencing burnout [6]. Among healthcare professionals, burnout prevalence remains particularly high because of excessive workload, long working hours, workplace violence, inadequate organizational support, and chronic job stress [7]. The other study found that 56% of illness‐related absences from work and 44% of all job‐related health issues were caused by depression and anxiety at work [2]. Despite there is little information available about the prevalence, severity, and determinants of WRS in this occupational category because the majority of the evidence currently available focuses on other types of healthcare workers in Ethiopia [8].

The study reported that WRS is attributed by a number of cleaning service respondents to a high workload, feeling exhausted or dizzy, bringing personal matters to work, and being offended by remarks made by coworkers [9]. WRS may also be impacted by workload, lengthy workdays, rotating or work shifts, working units, insufficient staffing, poor organizational support, job unhappiness, restricted autonomy, inadequate resources, interpersonal problems, and work‐family obligations. Consequently, while evaluating WRS within this underrepresented healthcare workforce, these attributes should be taken into account [10].

Although research on WRS has largely focused on clinical professionals such as nurses and physicians, support staff, particularly cleaners, remain underrepresented in occupational health research [1]. WRS among healthcare professionals is more prevalent in low‐ and middle‐income countries (LMICs) due to high patient loads, poor occupational health systems, insufficient staffing, and a lack of safety equipment. However, there is still a dearth of empirical research that focuses particularly on hospital cleaners because most studies look at nurses or other healthcare professionals and frequently classify cleaners as support staff without doing independent analysis [11]. Another important gap is the lack of standardized measurement tools for assessing WRS among non‐clinical hospital staff. Most studies in LMICs use adapted generalized stress questionnaires that have not been validated for cleaners' working conditions, limiting comparability and weakening evidence synthesis [10, 12].

The majority of Ethiopian research on hospital cleaners has focused on ergonomic and physical risks rather than the effects of psychological stress. For instance, research on the co‐occurrence of occupational outcomes among cleaners in eastern Ethiopia has only partially taken WRS into account as a secondary variable [12]. In a similar vein, studies conducted in Addis Ababa have revealed a significant frequency of occupational outcomes linked to WRS among cleaners, but WRS was regarded as a contributing factor rather than the main consequence [13]. Additionally, there is little cross‐national comparative data that could be used to pinpoint structural stressors in comparable economic environments [14].

Contextually, WRS among hospital cleaners in Ethiopia, notably the east of the country, has not been adequately investigated despite their critical role in maintaining sanitation and lowering infection risks in tertiary hospitals. In addition, workload, extended working hours, shift patterns, supervision, job demands, occupational dangers, and infection‐prevention obligations have received little attention for cleaners in tertiary hospitals. These elements may have a significant impact on their performance, safety, and occupational health. As a result, cleaners might continue to be underrepresented in psychological support services, occupational health programs, and workplace initiatives meant to lower stress and enhance worker well‐being.

So that there is a significant information gaps among cleaners in eastern Ethiopian tertiary hospitals, so that targeted cross‐sectional research is needed to determine the prevalence of WRS and its associated factors among these groups in order to provide evidence for just occupational health regulations and interventions. The Supporting Information Figure 1 (Figure S1) shows how the whole study framework was organized around key components that collectively form the research's conceptual framework. The study has three key messages for the readers, which are supplemented as Supporting Information Figure 2 (Figure S2).

## 2 Methods

### 2.1 Study Description and Period

The study was conducted in eight government hospitals in eastern Ethiopia from May to August 2023. The study defined eastern Ethiopia as the entire Harar area, the federal administrative city of Dire Dawa, the Oromia region, and a portion of Somalia. Eight government acute care centers participated in the inquiry. Since they were chosen at random from 14 hospitals, each inspection region had an equal chance (e.g., two hospitals were assigned to one area). Due to their workload and working characteristics, this study exclusively includes general hospitals, referral hospitals, and university teaching hospitals. Thus, Jogula General Hospital (JGH) and Haramaya University Hiwot Fana Comprehensive Specialized Hospital (HU‐HFCSH) are two of the medical institutions in the Harari region. Similarly, Chiro General Hospital (CGH) and Bisidimo General Hospital (BGH) were chosen among the State Health Facilities of Oromia Regional State. Dilchora Referral Hospital (DRH) and Sabian General Hospital (SGH), two medical facilities in Dire Dawa Federal Administration City, were included. Ultimately, two hospitals in the Somali Regional State were selected: Jigjiga University Sheik Hassan Yabare Referral Hospital (JURH) and Karamara General Hospital (KGH). The study areas for the assessment of the prevalence and predictors of work‐related stress among cleaners in tertiary hospitals in eastern Ethiopia showed in Figure 1.

**FIGURE 1:**
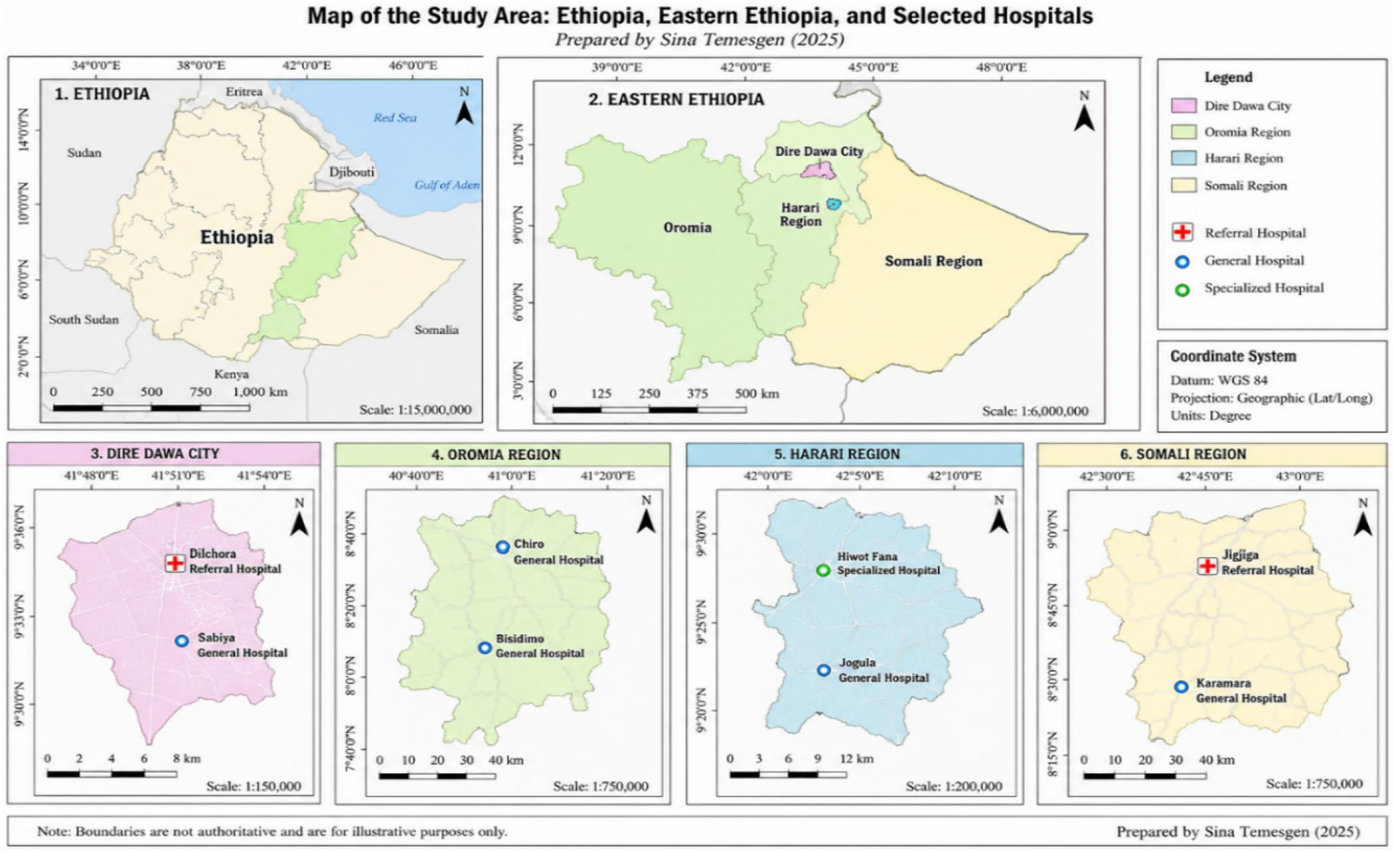
Map of the study areas for the assessment of the prevalence and predictors of work‐related stress among cleaners in tertiary hospitals in eastern Ethiopia, 2025.

### 2.2 Design of the Study

A hospital‐based cross‐sectional study was used to provide the prevalence of job stress among hospital cleaners in eight government hospitals in eastern Ethiopia between May and August 2023.

### 2.3 Studied Populations

The source population for this study comprised all hospital cleaners employed in government‐owned healthcare facilities across the eastern region of Ethiopia. The study participants included all cleaners recruited and officially employed by respective governmental health agencies within the same geographic area. Thus, the study considered cleaners working in public hospitals throughout eastern Ethiopia, ensuring full inclusion of eligible staff from each institution without exclusion based on department or facility, thereby providing a comprehensive representation of the target population.

### 2.4 Criteria for Inclusion and Exclusion

In this study, all cleaning staff employed at specific public hospitals were included. As a result, these cleaners are responsible for keeping the patient wards and restrooms clean. Moreover, individuals who have served as cleaners, garbage collectors, or empties in selected hospitals for over a month, regardless of whether they are on contract or permanent contracts, were chosen for the survey. However, the research did not take into account cleaners stationed in the administration building, cafeteria, and gardens, nor those who are on annual, sick, or maternity leave.

### 2.5 Sample Size Calculation

The sample size required for the study entitled “Job‐stress among hospital cleaners” was calculated using a single population proportion formula. It was done by considering an estimated prevalence of work‐related stress to be 45.2% [15], from the study conducted in Bahir Dar Textile Factory, North‐West Ethiopia, a 5% margin of error, a 95% confidence interval, and a 10% non‐response rate, which became 381. To ensure adequate representation across the study regions, a design effect of 2.0 was applied, resulting in a required sample size of 762. Because this number was very close to the total number of eligible hospital cleaners working in the eight selected hospitals (*n* = 741), and all eligible participants were accessible and manageable, the study included every eligible cleaner. Therefore, the study was conducted as a census of the target population rather than sampling a subset.

### 2.6 Methods Sampling Procedures

Eight public hospitals were randomly selected from a total of 14 public hospitals located in four regions of eastern Ethiopia using a lottery process. Two hospitals were selected from a single region to ensure that the sample is typical of the locations and to boost the response rate, following. Banerjee and Duflo [16] Justification. In light of this, 176 SWs were included from the following hospitals: 41 from Jogula General Hospital, 32 from Sabian General Hospital, 34 from Bisidimo General Hospital, 42 from Chiro General Hospital, 128 from Dilchora Referral Hospital, 225 from Jigjiga University Sheik Hassen Yabre Referral Hospital, and 63 from Karamara General Hospital.

### 2.7 Variables of the Study

There are two main types of study variables: independent variables, which the researcher alters to see their effects, and dependent variables, which measure job‐related stress to examine how they change in response to the independent variable. In this research, the dependent variable was work‐related stress resulting from the working conditions in hospitals. The measurement tool utilized was a series of Boolean logical questions and answers: YES [1] and NO [0], adapted [17, 18]. The total score obtained from all questionnaire items was summed for each participant and classified into two categories. Participants with a total score greater than or equal to the predetermined cut‐off value (mean) were classified as having work‐related stress (Yes), whereas those with scores below the cut‐off values were classified as not having work‐related stress (No). The primary outcome variable was dichotomous, which was used be analyzed using binary logistic regression to identify factors associated with work‐related stress. The experience of work‐related stress symptoms was rated by YES [1] and NO [0], adapted from [19].

While, the independent variables are sociodemographic characteristics, behavioral factors, psychosocial factors, organizational factors (workload, shift work, and working hours), and ergonomic/work environment factors, which may influence work‐related stress [20]. Socio‐demographic characteristics included age, sex, educational status, marital status, monthly income, job classification, and years of work experience. Personal factors comprised participants' knowledge, attitudes, and practices related to occupational health and safety. Institutional factors included the availability of personal protective equipment (PPE), access to work‐related training, and the implementation of infection prevention and control (IPC) practices within the workplace. Behavioral factors encompassed alcohol consumption, cigarette smoking, khat chewing, and sleep disorders. Work‐related factors included workload, working hours, workplace environmental conditions, social recognition, and other job‐related stressors. Each of these factors was assessed using Boolean logic, where responses are designated as YES [1] and NO [0], based on a preceding study [21].

### 2.8 Methods and Assessment Tools

The Copenhagen Psychosocial Questionnaire II (COPSOQ‐II) [22] and a previously modified version of the instrument [23] were used to assess work‐related stress among cleaners in tertiary hospitals. In order to adapt the questionnaire to the local context and occupational features of hospital cleaners, experts in public health and occupational health examined its suitability, relevance, and intelligibility. Items that were unrelated to the study group were altered or removed, while pertinent items from previous research were included. A pretest of the amended questionnaire was administered to a group of hospital cleaners who were not involved in the main study in order to assess the questions' appropriateness, clarity, and comprehensibility. Based on pretest feedback, ambiguous or unclear items were amended prior to the final data collection. The internal consistency of the updated questionnaire was evaluated during the pre‐test utilizing nine items and Cronbach's alpha coefficient. The study produced a Cronbach's alpha of 0.81, which suggests that the items accurately measured the target construct of WRS and indicates strong internal consistency.

The components of work‐related stress physical indicators, which included muscular pain, tremors, palpitations, diarrhea, sweating, respiratory distress, dizziness, indigestion, headache, backache, poor sleep, loss of libido, elevated heart rate, elevated blood pressure, and dry mouth or throat were assessed by Boolean responses (YES [1]/NO [0]), and classified each as low and severe according to the earlier study [24]. Lastly, by summed up the components of WRS physical indicators, the final outcomes, work‐related stress as severe if higher than mean scores, and low if lower than mean scores, adapted from previous study conducted in the country [8]. Boolean responses (YES [1]/NO [0]) were used to evaluate behavioral aspects, such as sleep difficulties, heavy alcohol intake, chewing Khat, and cigarette smoking, which were modified from a prior study [25]. Adapted from earlier studies [17, 20]. Safety variables were defined as workplace settings that safeguard employees' well‐being, such as occupational health and safety (OHS) measures, controlled daily working hours, and a manageable workload.

### 2.9 Collection Process and the Participants

A total of eight data collectors participated in the data collection process. The team comprised two individuals holding MSc degrees in Environmental Health, two with MSc degrees in Occupational Health and Safety, and four with MPH degrees in Public Health. All data collectors were carefully selected based on their relevant academic background and experience to ensure high‐quality and reliable data collection. In addition, four supervisors were assigned to oversee and coordinate the data collection activities. These supervisors provided close technical support, ensured adherence to the study protocol, and monitored data quality across the eight public hospitals included in the study. Their role also involved routine field supervision, clarification of procedural issues, and verification of completed questionnaires to maintain consistency and completeness of the collected data.

### 2.10 Procedures of Data Collection in Sites

Following the approval of the study by public hospitals, a team of eight data collectors and four supervisors was assigned to the collection locations. The data collectors were briefed about the three shifts for hospital cleaners. Over the course of the year, the duration and frequency of the SWs' shifts can vary from 1 month to 3 months, depending on the specific hospital. Within a 24‐h period, hospital cleaners were divided into three work shifts. The first shift ran from 7:00 a.m. to 12:00 p.m. (5 h), the second shift ran from 1:00 p.m. to 5:00 p.m. (4 h), and the third shift ran from 5:00 p.m. to 7:00 a.m. (14 h). The entire 24‐h workday was thus covered by the three shifts taken together. This group works according to their schedule for 2 consecutive days, followed by 2 days off. Consequently, the questionnaires for shift 1 were distributed during the morning hours (9:00–10:00). The interview was conducted for shift 2 in the afternoon, from 3:00 to 4:00. Since they don't work 24 h a day, if they work at night, the identical procedure was used for shift 3 on 2 days.

### 2.11 Data Quality Management

The initial version of the questionnaires was created for quality management after conducting a literature review on work‐related stress. To ensure high data quality, standardized questionnaires in English were formulated for each target group, incorporating both multiple‐choice and open‐ended questions. Following local preferences, these materials were then translated into three regional languages attached as Supplementary File (Supporting File). Subsequently, eight data collectors and supervisors with relevant experience were appointed. Additionally, both managers and data collectors underwent thorough training to effectively engage with respondents and complete the surveys, especially those focusing on raising awareness about occupational or job‐related stress. The final objective is to carry out a study on the reliability and validity of quantitative data using appropriate terminology, concepts, and statistical models as indicated in previous research [22, 26]. To ensure the clarity and effectiveness of the questionnaires and to avoid confusion during data collection and measurement, a pretest study involving 5% of the employees at Haramaya General Hospital (HGH) was conducted. Sixth, the internal consistency of the revised work‐related stress questionnaire was assessed using Cronbach's alpha [27]. The overall alpha coefficient was **0.81**, exceeding the acceptable threshold of 0.70, indicating strong internal consistency and adequate reliability for assessing work‐related stress in the study population. Seventh, information from those whose data quality was subpar was immediately eliminated throughout the screening phase.

### 2.12 Data Analysis

First, Epi‐Data 3.1 was used to double‐enter, validate, and sanitize the coded quantitative data. The data were then exported using Stata 17MP. There were two statistical analyses performed. First, mean, median, and standard deviations (continuous variables) and frequencies, proportions, and percentages (categorical variables) were subjected to descriptive statistics. Second, a statistical analysis was carried out to determine the link between independent factors and dependent variables. Binary logistic regression was used in the bivariate and multivariate stages of the study since the data were binomial. Independent variables with a binary logistic regression analysis *p*‐value of 0.20 were considered candidates for the multivariate analysis. Only variables with a p‐value of 0.05 were deemed statistically significant at model 0.20, and they were eliminated in a stepwise backward procedure. The 95% confidence interval (CI) for the crude odds ratio (COR) and adjusted odds ratio (AOR) was displayed. Determinants with an AOR and a p‐value of 0.05 (CI:95%) were then reported. Two‐tailed *p*‐values are used to test whether there is a significant difference in either direction between variables. With a cut‐off point of less than 10, the variance inflation factor (VIF) calculates the relationship between the variance of one independent variable and the others [28]., was used to test for multicollinearity. In addition, Hosmer and Lemeshow's (HL) goodness‐of‐fit test was employed for regression model fit. The HL with a small Chi‐square value with a *p*‐value more than 0.05) was approved according to [28] interpretation. The overall data analysis, reporting and interpretation of statistics values were done accordance with [29] guideline.

### 2.13 Ethical Considerations

Ethical approval for this study was obtained from the Institutional Health Research Ethics Review Committee (IHRERC) of the College of Health and Medical Sciences, Haramaya University, Ethiopia (Reference No. IHRERC/064/2023). The study was conducted in accordance with internationally accepted ethical principles for research involving human participants, including the principles outlined in the *Declaration of Helsinki*. Prior to data collection, formal permission was obtained from the administrations of the participating hospitals. The purpose, objectives, procedures, potential benefits, and possible risks of the study were clearly explained to all eligible participants. Participants were also informed of their rights to ask questions, decline participation, or withdraw from the study at any stage without any penalty, loss of benefits, or consequences to their employment or access to services.

Written informed consent was obtained voluntarily from each participant before enrollment in the study. Participation was entirely voluntary, and no coercion or undue influence was used during the recruitment process. Participants were assured that refusing to participate or discontinuing participation would not affect their professional responsibilities or relationship with their respective institutions. Strict measures were implemented to ensure confidentiality and privacy throughout the research process. No personally identifiable information, such as participants' names or other unique identifiers, was collected on the questionnaires. Instead, each questionnaire was assigned a unique identification code, and all collected data were anonymized (or pseudonymized where appropriate) before analysis. The coding system ensured that individual participants could not be identified from the research data.

All completed questionnaires and electronic datasets were securely stored in password‐protected computers and locked cabinets that were accessible only to the principal investigators and authorized research personnel. The data were used exclusively for scientific and academic purposes and were not shared with unauthorized individuals or institutions. The findings are presented in aggregate form, ensuring that no participant, hospital, or department can be individually identified from the published results. The study adhered to the principles of respect for persons, beneficence, non‐maleficence, and justice throughout the research process. Every reasonable effort was made to minimize potential risks while maximizing the scientific and public health value of the research. The confidentiality, dignity, rights, and welfare of all participants were protected throughout the study, consistent with the ethical standards of the Declaration of Helsinki and the ethical guidelines governing human subject research.

## 3 Results

### 3.1 Participants' Characteristics

Of the 741 public hospital cleaners approached, 679 participated in the study, giving a response rate of 91.63%. Of the participants, 672 (98.97%) were female and seven (1.03%) were male. The average age of the participants was 34.28 ± 7.46 years, with 329 (48.45%) being under 34 and 350 (51.55%) being above 34. In terms of marital status, 467 (68.78%) were married, 76 (11.19%) were widowed or divorced, and 136 (20.03%) were single. With a mean educational level of 6.80 ± 2.51 years, 413 (60.82%) had completed ≥ Grade 6, while 266 (39.18%) had completed. A mean monthly income of 19,667 ± 342 ETB was reported, with 207 (30.49%) participants earning ≥ 1667 ETB and 472 (69.51%) earning less than 1967 ETB. With a mean work service of 6.72 ± 6.52 years, 456 (67.16%) participants had worked for less than 6 years, while 223 (32.84%) had worked for more than 6 years. The majority of participants were married, at least 34 years old, female, had completed at least Grade 6, and had worked for fewer than 6 years (Table 1).

**TABLE 1:** Presented sociodemographic status among selected public hospitals' cleaners in eastern Ethiopia, 2025 (*N* = 679).

Variables	Group	Frequency	Percentage	Mean & std. deviation
Sex	Female	672	98.97	
	Male	7	1.03	
Age of participants	< 34	329	48.45	34.28 ± 7.46
	≥ 34	350	51.55	
Marital status	Single	136	20.03	
	Married	467	68.78	
	Separated	76	11.19	
Educational status	< Grade 6	266	39.18	
	≥ Grade 6	413	60.82	
Working shifts per a month	1	338	49.78	
	2	242	35.64	
	3	99	14.58	
Income monthly salary (ETB)	≥ 1667 ETB	207	30.49	19,667 ± 342
	< 1967 ETB	472	69.51	
Work service	< 6 years	456	67.16	6.72 ± 6.52
	≥ 6 years	223	32.84	

Key: Ethiopia's civil officials' salary scale: These levels were classified according to the national evaluation and Grading [JEG, 2019] of Ethiopia's civil officials' wage system, so that * indicates minimum level (I) of salary in Ethiopia (1100 ETB); **Level V, which was the maximum level of salary for cleaners during study period. During study period: 1 dollar equaled 55.45 ETB, September 2023.

## 4 Behavioral and, Safety Environmental Factors

Of the 679 participants, 272 (40.06%) did not report having a sleep issue or disruption because of their job, whereas 407 (59.94%) did. 227 people (33.43%) said they have a workload at the moment, while 452 people (66.57%) did not. Of the participants, 380 (55.96%) reported working 8 h or less, whereas 299 (44.04%) reported working more than 8 h. Of the individuals, 519 (76.44%) did not chew Khat, whereas 160 (23.56%) reported doing so. In a similar vein, 560 people (82.47%) did not now smoke, whereas 119 (17.53%) smoked. Regarding occupational health and safety services, 406 participants (59.79%) rated adequate services, whereas 273 participants (40.21%) reported poor services.

In terms of social elements and job‐related satisfaction, 182 (26.80%) individuals were dissatisfied with their current position, compared to 497 (73.20%) who were. Of the participants, 630 (92.78%) expressed satisfaction with the work environment, whereas 49 (7.22%) expressed dissatisfaction. 385 (56.70%) of the individuals reported social recognition, while 294 (43.30%) did not. In terms of the frequency of supervision, 467 individuals (68.78%) reported receiving supervision at least once a month (≥ monthly), whereas 212 participants (31.22%) reported having supervision less than once a month. Overall, these results show significant sleep disturbances at work, long workdays, worries about occupational safety, and disparities in the participants' job satisfaction, social recognition, workload, and supervision (Table 2).

**TABLE 2:** Work‐related behavioral, safety, and environmental factors among neglected work forces (hospital cleaners) in eastern Ethiopia (*N* = 679).

Variable	Category	Frequency (*n*)	Percentage (%)
Sleep disorder/disturbance due to work	Yes	407	59.94
	No	272	40.06
Currently have workload	Yes	227	33.43
	No	452	66.57
Currently work more than 8 h	Yes	299	44.04
	No	380	55.96
Currently chewing Khat	Yes	160	23.56
	No	519	76.44
Currently smoke	Yes	119	17.53
	No	560	82.47
Inadequate of occupational and health safety service	Yes	273	40.21
	No	406	59.79
Satisfied with current job	Yes	497	73.20
	No	182	26.80
Satisfied with work situation	Yes	630	92.78
	No	49	7.22
Social recognition	Yes	385	56.70
	No	294	43.30
Supervision frequency	< Monthly	212	31.22
	≥ Monthly	467	68.78

### 4.1 Prevalence and Symptom of Work‐Related Stress

Figure 2 presents that out of the 679 employees, 198 (29.16%; 95% CI: 25.87%–32.69%) said they had experienced severe work‐related stress at work in the previous 12 months. This significant percentage may have a detrimental impact on employees' motivation, focus, productivity, and job performance, which could eventually lower the standard and effectiveness of medical services. On the other hand, 481 employees (70.84%; 95% CI: 67.31%–74.13%) reported having low work‐related stress during the same time period, suggesting that the majority of workers were able to work without self‐reporting occupational stress.

**FIGURE 2:**
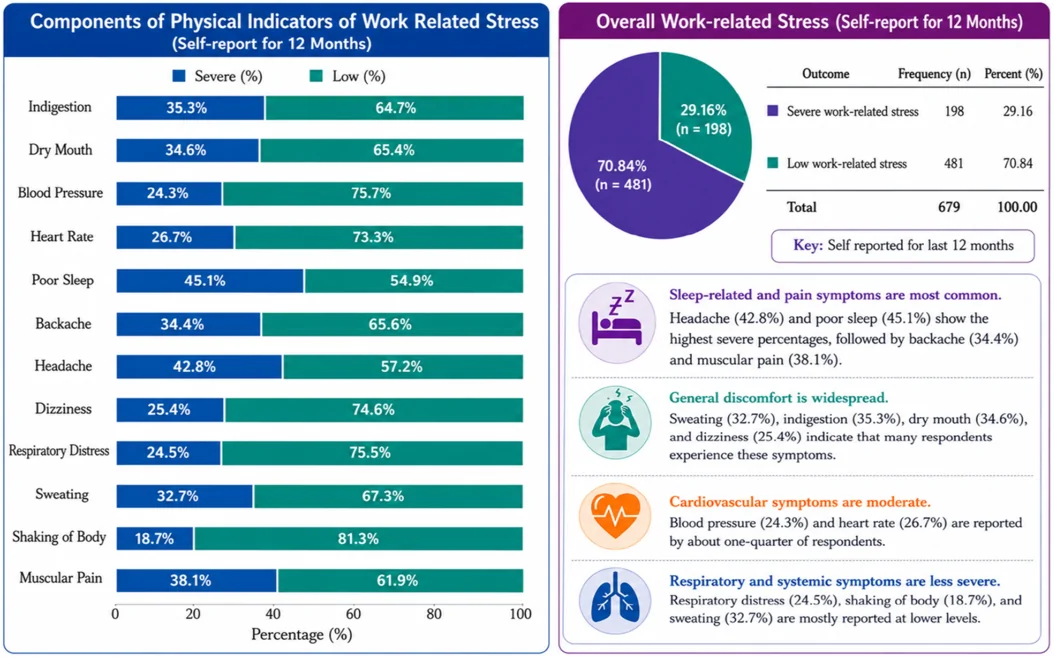
The common experienced symptoms among neglected work forces (hospital cleaners) in eastern Ethiopia (*N* = 679).

Specifically, among the 198 (29.16%) hospital cleaners who experienced with severe work‐related stress, the distribution of stress‐related health symptoms during the previous month indicated that physical and psychological complaints were common. In terms of specific symptoms, headache (42.8%), severe muscular pain (38.1%), and disturbed sleep (45.1%) were the most commonly reported symptoms. Additionally, 34.4% of cleaners reported having severe back pain, while 35.3% reported having severe indigestion and 34.6% reported having severe dry mouth. A total of 32.7% of interviewees reported severe sweating, suggesting that a sizable percentage of cleaners had overall physical discomfort for the last working year.

The finding also revealed that heart‐rate variations were reported by 26.7% of cleaners, whereas contracted high blood pressure‐related symptoms were reported by 24.3%. Cardiovascular‐related symptoms were reported less frequently. Similarly, 25.4% of respondents reported feeling lightheaded, and 24.5% reported experiencing respiratory discomfort. The least common symptom (18.7%) was shaking of the body. Overall, the results show that around three out of 10 cleaners had WRS during the previous 12 months, with headaches, muscular discomfort, and sleep difficulties being the most commonly reported symptoms. These results imply that a wide range of physical symptoms, especially those related to sleep, pain, and overall discomfort, were associated with WRS (Figure 2).

### 4.2 Binary Logistic Regression Analysis

In the COR analysis, 25 variables are evaluated. Eighteen variables qualified for the multivariable analysis based on the criterion *p* < 0.20. Accordingly, the initial model found that workload was found to have the strongest positive crude association with severe work‐related stress(WRS) (COR = 17.89, 95% CI: 11.85–27.00, *p* < 0.001), followed by sex (COR = 15.00, 95% CI: 1.79–25.42, *p* < 0.012), smoking (COR = 14.59, 95% CI: 9.05–23.54, *p* < 0.001), chewing khat (COR = 7.75, 95% CI: 5.24–11.46, *p* < 0.001), and heavy alcohol consumption (COR = 6.75, 95% CI: 3.82–11.93, *p* < 0.001), inadequate of occupational health and safety (OHS) service (COR: 1.76, 95% CI: 1.26–2.47, *p* < 0.001) were significantly increase the crude odds of WRS.

Also, job dissatisfaction (COR = 3.99, 95% CI: 2.46–6.46, *p* < 0.001), education ≥ Grade 6 (COR = 3.64, 95% CI: 2.46–5.40, *p* < 0.001), lack of social recognition (COR = 3.05, 95% CI: 2.11–4.40, *p* < 001), infrequent supervision (COR = 2.87, 95% CI: 2.03–4.06, *p* < 0.001), working > 8 h/day (COR = 2.001), and sleep disorder (COR = 2.39, 95% CI: 1.70–3) were significantly increase the crude odds of severe work related stress among cleaners. Conversely, contract employment (COR = 0.36, 95% CI: 0.14–0.90, *p* = 0.028), working shift 2 (COR = 0.32, 95% CI: 0.22–0.48, *p* < 0.001), shift 3 (COR = 0.28, 95% CI: 0.16–0.50, *p* < 0.001), and ≥ 6.72 years of work experience were all related to lower the severe work‐related risks of stress (Table 3).

**TABLE 3:** Binary logistic regression analysis model for associated factors of work‐related stress among neglected work forces (hospital cleaners) in eastern Ethiopia (*N* = 679).

Variable	Category	Severe stress: 198 (29.16%)	Low stress: 481 (70.84%)	COR (95% CI)	*p*‐value
Presence of workload	No (ref.)	46 (6.8)	406 (59.8)	1	
	Yes	152 (22.4)	75 (11.0)	17.89 (11.85–27.00)	< 0.001**
Sex	Female (ref.)	192 (28.3)	480 (70.7)	1	
	Male	6 (0.9)	1 (0.1)	15.00 (1.79–25.42)	0.012**
Smoking	No (ref.)	106 (15.6)	454 (66.9)	1	
	Yes	92 (13.5)	27 (4.0)	14.59 (9.05–23.54)	< 0.001*
Khat chewing	No (ref.)	96 (14.1)	423 (62.3)	1	
	Yes	102 (15.0)	58 (8.5)	7.75 (5.24–11.46)	< 0.001*
Heavy alcohol use	No (ref.)	155 (22.8)	462 (68.0)	1	
	Yes	43 (6.3)	19 (2.8)	6.75 (3.82–11.93)	< 0.001*
Current job satisfaction	Yes (ref.)	176 (25.9)	321 (47.3)	1	
	No	22 (3.2)	160 (23.6)	3.99 (2.46–6.46)	< 0.001*
Social recognition	Yes (ref.)	148 (21.8)	237 (34.9)	1	
	No	50 (7.4)	244 (35.9)	3.05 (2.11–4.40)	< 0.001*
Supervision frequency	≥ Monthly (ref.)	103 (15.2)	364 (53.6)	1	
	< Monthly	95 (14.0)	117 (17.2)	2.87 (2.03–4.06)	< 0.001*
Work > 8 h	No (ref.)	75 (11.0)	305 (44.9)	1	
	Yes	123 (18.1)	176 (25.9)	2.84 (2.02–4.00)	< 0.001*
Sleeping disorder	No (ref.)	89 (13.1)	318 (46.8)	1	
	Yes	109 (16.1)	163 (24.0)	2.39 (1.70–3.35)	< 0.001*
Inadequate of OHS service	No (ref.)	99 (14.6)	307 (45.2)	1	
	Yes	99 (14.6)	174 (25.6)	1.76 (1.26–2.47)	< 0.001*
Work situation satisfaction	Yes (ref.)	190 (28.0)	440 (64.8)	1	
	No	8 (1.2)	41 (6.0)	2.21 (1.02–4.81)	0.045**
Monthly income salary	≥ 1967 (ref.)	39 (5.7)	168 (24.7)	1	
	< 1966	159 (23.4)	313 (46.1)	2.19 (1.47–3.26)	< 0.001*
Work experience	< 7 years (ref.)	106 (15.6)	350 (51.5)	1	
	≥ 7 years	92 (13.5)	131 (19.3)	0.43 (0.31–0.61)	< 0.001*
Education	< Grade 6 (ref.)	39 (5.7)	227 (33.4)	1	
	≥ Grade 6	159 (23.4)	254 (37.4)	3.64 (2.46–5.40)	< 0.001*
Employment type	Permanent (ref.)	188 (27.7)	472 (69.5)	1	
	Contract	10 (1.5)	9 (1.3)	0.36 (0.14–0.90)	0.028**
Marital status	Married (ref.)	140 (20.6)	327 (48.2)	1	
	Single	30 (4.4)	106 (15.6)	0.66 (0.42–0.94)	0.072***
	Separated	28 (4.1)	48 (7.1)	1.36 (0.82–2.26)	0.231
Working shift	Shift 1 (ref.)	138 (20.3)	200 (29.5)	1	
	Shift 2	44 (6.5)	198 (29.2)	0.32 (0.22–0.48)	< 0.001*
	Shift 3	16 (2.4)	83 (12.2)	0.28 (0.16–0.50)	< 0.001*

Legend: Two‐tailed *p*‐values are used to test difference with

* *p*‐value < 0.001 → Highly statistically significant;

** *p*‐value < 0.05 → Statistically significant;

*** *p*‐value < 0.20 → Statistically significant at Binary analysis.

### 4.3 Multivariable Logistic Regression Analysis

The multivariable logistic regression analysis of occupational stress identified 10 variables that were statistically significant. The final model found that cleaners who reported having a workload were 10.23 times more likely to experience severe work‐related stress than those who did not (AOR = 10.23, 95% CI: 6.16–16.98; *p* < 0.001). The model found that the severity of WRS was 6.64 times greater in smokers than in non‐smokers (AOR = 6.64, 95% CI: 3.53–12.51; *p* < 0.001) and 4.07 times higher in khat chewers than in non‐chewers (AOR = 4.07, 95% CI: 2.34–7.09; *p* < 0.001). Stress was 2.67 times more likely to occur among employees who were dissatisfied with their current position than in those who were (AOR = 2.67, 95% CI: 1.24–5.76; *p* = 0.012). Likewise, less than monthly supervision (AOR = 1.63, 95% CI: 1.01–3.06; *p* = 0.030), lack of social recognition (AOR = 3.11, 95% CI: 1.63–5.92; *p* < 0.001) were associated with increase of WRS.

Meanwhile, the odds of severe work‐related stress were 2.18 times higher for those who reported sleeping difficulties than for those who did not (AOR = 2.18, 95% CI: 1.27–3.71; *p* = 0.004). Stress was also substantially correlated with inadequate of OHS services (AOR = 1.64, 95% CI: 1.16–2.83; *p* = 0.043). Conversely, those with more than 7 years of working experience were 56% less likely to severe work‐related stress than those with less than 6.72 years (AOR = 0.44, 95% CI: 0.26–0.75; *p* = 0.003). Similarly, the odds of severe work‐related stress were 71% lower for workers with ≥Grade 6 education than for those with less than sixth grade (Table 4).

**TABLE 4:** Multivariable logistic regression analysis model for associated factors of work‐related stress among neglected work forces (hospital cleaners) in eastern Ethiopia (*N* = 679).

Variable	Category	Severe stress: 198 (29.16%)	Low stress: 481 (70.84%)	AOR (95% CI)	*p*‐value
Presence of workload	No (ref.)	46 (6.8)	406 (59.8)	1	
	Yes	152 (22.4)	75 (11.0)	10.23 (6.16–16.98)	< 0.001*
Smoking	No (ref.)	106 (15.6)	454 (66.9)	1	
	Yes	92 (13.5)	27 (4.0)	6.64 (3.53–12.51)	< 0.001*
Khat chewing	No (ref.)	96 (14.1)	423 (62.3)		
	Yes	102 (15.0)	58 (8.5)	4.07 (2.34–7.09)	< 0.001*
Current job satisfaction	Yes (ref.)	176 (25.9)	321 (47.3)	1	
	No	22 (3.2)	160 (23.6)	2.67 (1.24–5.76)	0.012**
Social recognition	Yes (ref.)	148 (21.8)	237 (34.9)	1	
	No	50 (7.4)	244 (35.9)	3.11 (1.63–5.92)	< 0.001*
Supervision frequency	≥ Monthly (ref.)	103 (15.2)	364 (53.6)	1	
	< Monthly	95 (14.0)	117 (17.2)	1.63 (1.01–3.06)	0.030**
Sleeping disorder	No (ref.)	89 (13.1)	318 (46.8)		
	Yes	109 (16.1)	163 (24.0)	2.18 (1.27–3.71)	0.004**
Inadequate of OHS service	No (ref.)	99 (14.6)	307 (45.2)	1	
	Yes	99 (14.6)	174 (25.6)	1.64 (1.16–2.83)	0.043**
Work experience	< 7 years (ref.)	106 (15.6)	350 (51.5)		
	≥ 7 years	92 (13.5)	131 (19.3)	0.44 (0.26–0.75)	0.003**
Education	< Grade 6 (ref.)	39 (5.7)	227 (33.4)		
	≥ Grade 6	159 (23.4)	254 (37.4)	0.29 (0.16–0.54)	< 0.001*

Legend: Two‐tailed *p*‐values are used to test whether there is at significant difference with

* *p*‐value < 0.001 → Highly statistically significant;

** *p*‐value < 0.05 → Statistically significant.

## 5 Discussion

The current study aimed to assess work stress and associated factors among hospital cleaners in Ethiopia. Accordingly, the study population was predominantly female, indicating a strongly gender‐skewed workforce in the study setting. The majority of study participants were with a mean age of 34.28 years (± 7.46), the majority of the participants appeared to be in the economically active age group. Marital status revealed that workers who were widowed or divorced made up a smaller percentage, while married participants made up the largest group, followed by single participants. This pattern suggests that a large number of cleaners had family obligations in addition to their work‐related commitments, which may have an impact on their exposure to occupational stress and work‐life balance. In general, the age and marital features point to a workforce that is comparatively mature and has significant social and familial obligations. These traits are crucial when taking into account the psychological and occupational factors that could affect hospital cleaners' work‐related stress in the study scenario.

The participants' mean educational attainment was close to 7 grades (± 2.51) in terms of occupational and educational parameters. While a sizable percentage had received schooling below grade 6, more than half had finished grade 6 or higher. This suggests that the workforce's educational attainment was typically low to moderate. The average employment experience was acceptable 7 years (± 6.52), indicating a wide range of work exposure. While some participants had worked for 6 or more years, the majority had fewer than 6 years. Some employees' comparatively long employment histories may be a sign of extended exposure to working circumstances and pressures. Therefore, disparities in occupational knowledge, ability to adjust to workplace expectations, and susceptibility to job‐related stress among hospital cleaners may be explained by educational attainment and work experience.

About half of the participants worked one shift per month, a sizable portion worked two shifts, and a smaller group worked three shifts, according to work organization and socioeconomic characteristics. The variance in shift patterns suggests that hospital cleaners have different work schedules and job distributions. Fatigue and occupational stress may be exacerbated by working several extended shifts, which may limit possibilities for proper rest and recuperation. Additionally, the majority of participants earned less than the declared income criterion, with a mean monthly wage of roughly 1967 ETB (± 342). This indicates that the research population generally has minimal financial resources. Low pay and inconsistent work schedules may put cleaners under more psychological and financial strain, which may have an impact on their general occupational well‐being and stress levels.

### 5.1 Descriptive Behavioral and Safety Analysis

In terms of behavioral and safety‐related issues, a significant percentage of participants reported sleep disruption connected to work, suggesting that occupational pressures may impact workers' rest and recuperation [23, 25]. While the majority of participants did not perceive workload as a problem, a smaller percentage reported having a present workload. In terms of working hours, almost half of the participants put in more than 8 h a day, indicating that longer workdays were somewhat typical. The majority of interviewees stated that they neither currently smoke nor chew Khat. In terms of occupational health and safety services, a significant percentage reported poor services, while over half of the participants thought the services were adequate. These results point to a number of occupational and behavioral factors that could affect employees' well‐being and safety at work.

Regarding social and occupational aspects, the majority of participants reported being content with their present position, suggesting generally positive opinions of their work. There was also a high level of contentment with the workplace, with very few people expressing discontent. While a sizable minority of participants said they did not receive social recognition for their work, over half of the participants said they did. These results imply that there are still chances to improve recognition and assistance in the workplace, even though overall job satisfaction and opinions of the workplace were positive [17, 20]. Because it can affect workers' motivation, sense of worth, and dedication to their duties, social acknowledgment may be especially significant. Therefore, filling in recognized gaps may help create a more encouraging and fulfilling workplace.

In terms of supervision, a lesser percentage of participants said that supervision happened less frequently, although the majority reported receiving supervisory support at least once a month. Frequent supervision may offer chances for direction, performance evaluation, workplace problem detection, and reinforcement of occupational safety procedures, which is similar to the previous studies report [17, 20]. However, the existence of employees who receive less regular supervision raises the possibility that not all participants receive the same level of supervisory coverage. The results show both positive and negative behavioral, safety, and workplace situations overall. Substance abuse was indicated by fewer participants, although sleep disturbance and long work hours were significant issues. Job and environmental satisfaction were similarly favorable, as were perceptions of occupational health and safety services. However, disparities in supervision and social recognition point to areas where workplace support should be improved.

### 5.2 Descriptive Work‐Related Stress Analysis

The current study found that the prevalence of WRS among hospital cleaners in eastern Ethiopia was 29.16%. This finding demonstrates that they were accompanied by a wide range of physical symptoms, with strain and sprain complaints, autonomic manifestations, sleep disturbances, and headaches being the most prevalent. The high frequency of these symptoms underscores the substantial occupational health burden associated with work‐related stress in this workforce and highlights the need for effective workplace stress prevention and management interventions. The current percentage implies that there are numerous significant workplace problems, such as an excessive workload, unpleasant working circumstances, a lack of management, inadequate support, and disagreements between employees [30]. These issues have a detrimental effect on employee productivity, health, and well‐being, and raise absenteeism and turnover rates, as well as the risk of physical and mental health problems like depression, heart disease, and burnout [31].

When we compare the current finding (29.16%) with the previous studies, it is slightly higher than the 21.7% reported from Indonesia [2]; 13.4% reported from Vietnam [32]. But, it was lower than 44% [33]; 58.2% reported from Gondar, Ethiopia [34]; and 42.9% in Thailand [35]. One of the following causes or contributing variables could be the cause of the overall disparities. The participants' perceptions of danger as a result of their awareness of WRS could be the first explanation for the discrepancy [36]. The second explanation for the disparity could be attributed to institutional and environmental factors, which have a major impact on workers' exposure to workplace stress in industrialized and low‐middle‐income nations [37, 38]. The third rationale is that the research's methodology, which includes data collection instruments, sample size, study depth, and professional involvement, may have an impact on the results [39, 40]. The fourth explanation for the disparity is that other countries have distinct workplace safety, health laws, rules, and policies, which may further contribute to the substantial amount of WRS [41].

### 5.3 Final Model Analysis for Associated Factors of WRS

The multivariable logistic regression model found that work‐related stress (WRS) among hospital cleaners was found to be substantially associated with workload, behavioral traits, sleep issues, job satisfaction, social recognition, supervision, occupational health and safety (OHS) services, work experience, and educational status. The almost tenfold higher risks of WRS experienced by cleaners who reported a workload compared to those who did not show that excessive job demands can greatly increase psychological and physical strain. This is consistent with previous research on hospital cleaners in eastern Ethiopia, which discovered that while workload was associated with better occupational outcomes, excessive work hours and a lack of supervision also related to unpleasant working conditions [42, 43, 44, 45]. The physically taxing nature of cleaning job, time constraints, a lack of workers, and a lack of recovery chances may all contribute to the high correlation shown in this study. Workload, poor supervision, and bad working circumstances are all significant occupational risk factors, according to data from Ethiopian hospital cleaners.

The final model also found that WRS was also significantly influenced by behavioral and sleep‐related factors. The probabilities of experiencing stress were almost 6 and a half times greater for those who smoked cigarette and 4 times higher for those who chewed khat. These results corroborate other research mentioned in this paper, which suggests that substance abuse may be linked to occupational stress through poor psychological health, a diminished ability to cope, and unhelpful reactions to demands at work. According to this study, behavioral issues and occupational stress among hospital cleaners are highly correlated [12]. The previous study found that workers who suffer from behavioral addictions or disorders are likely to have higher levels of stress related to their jobs because these addictions often involve a constant need for stimulation or escape, can have negative consequences like poor performance or financial difficulties that affect work, resulting in job stress [46]. Along with this, one study reported that behavioral disorders or abnormalities can result from or worsen underlying issues like stress or other mental health conditions, all of which contribute to stress [47].

In a similar vein, the odds of WRS were more than twice as high for cleaners who reported sleeping issues as for those who did not. This result is in line with earlier studies conducted among eastern Ethiopian hospital cleaners, which found a high correlation between sleep disturbances and unfavorable occupational outcomes [43]. There may be a reciprocal relationship, with occupational stress interfering with sleep and insufficient or disturbed sleep impairing emotional control, focus, and ability to withstand pressures at work. The findings further demonstrate cleaners who were dissatisfied with their current jobs had approximately threefold higher odds of experiencing stress than those who were satisfied. This finding is consistent with the previous studies cited in this research, which indicate that job satisfaction can act as a protective factor by strengthening engagement, perceived support, and psychological well‐being [48, 49]. Evidence from Ethiopian workers likewise found that lack of job satisfaction was strongly associated with WRS, supporting the role of positive work experiences in reducing occupational stress.

Furthermore, the model found that cleaners who lacked social recognition had approximately threefold higher odds of WRS than those who received recognition. This finding is consistent with previous studies showing that poor social recognition and inadequate workplace support increase occupational vulnerability among hospital cleaners [10, 12]. Recognition from hospital staff and the wider hospital community may therefore enhance workers' sense of value, belonging, and organizational support, thereby strengthening their ability to cope with occupational demands.

WRS was also substantially correlated with OHS services and supervision, which was also explained by [45]. In addition. WRS was around 1.5 times more common among cleaners who were supervised less frequently than once a month than among those who were supervised at least once a month. This confirms earlier findings that regular supervision can offer direction, spot issues at work, enhance task organization, and bolster managerial and social support [44, 45].

According to studies conducted among cleaners in tertiary of hospital found in eastern Ethiopia, poor supervision was associated to negative work outcomes. Furthermore, the probabilities of WRS were close two‐fold greater among cleaners who reported receiving subpar OHS services. This result is in line with data from Addis Ababa that demonstrates the significance of organizational factors and occupational safety and health training in determining the well‐being of cleaners [8]. Also, the model found that inadequate OHS services can lower employees' trust that their employer is sufficiently safeguarding their health and safety and raise their perceived susceptibility to workplace risks.

It's interesting to note that greater educational attainment and more work experience seemed to protect against WRS. Compared to individuals with less than 7 years of experience, cleaners with 7 or more years of job experience were roughly 56% less likely to suffer stress. Similarly, compared to those with lesser educational attainment, those with Grade 6 or above education had almost 71% lower risk of WRS. Longer experience may have a protective impact since it increases one's familiarity with job demands, coping mechanisms, workplace processes, and confidence in handling obstacles at work. In same vein, higher levels of education may also improve employees' comprehension of workplace hazards, ability to communicate with managers, capacity for problem‐solving, and availability of helpful resources. These results imply that the psychological effects of hard cleaning tasks may be mitigated by personal ability and cumulative work experience. These findings and explanations are consistence with the previous studies reported from Indonesia [2], Vietnam [32], Ethiopia [34], and Thailand [35].

Overall, the results show that workload, behavioral and sleep‐related circumstances, organizational support, recognition, job satisfaction, and individual characteristics all interact to cause WRS among hospital cleaners. The necessity for programs that integrate workload management with occupational health promotion is highlighted by the particularly strong connections found for smoking, chewing khat, and workload. WRS may be decreased by regular supervision, sufficient OHS services, fair workload distribution, supportive management, acknowledgment of cleaners' contributions, and actions encouraging restful sleep and good coping. Evidence that organizational and psychosocial interventions targeting workload, social support, supervision, and workplace circumstances can enhance hospital cleaners' occupational well‐being supports these strategies.

### 5.4 Analysis of Policy Implications

The study identified a 29.16% prevalence of work‐related stress among hospital cleaners in eastern Ethiopia, with commonly reported moderate to severe symptoms, including dizziness, excessive sweating, muscular discomfort, and respiratory distress. These findings indicate that occupational stress among this workforce represents a meaningful public health and occupational health concern requiring structured responses across multiple levels of the health system. Therefore, from a policy perspective, the observed burden of work stress necessitates the integration of occupational mental health and psychosocial risk management into national and regional health and labor policies. Regulatory frameworks for healthcare institutions require strengthening to ensure systematic assessment of psychosocial hazards alongside physical risks in low‐wage hospital occupations. Policy direction at federal and regional levels becomes essential for embedding routine occupational health surveillance, workload regulation, and enforceable standards for worker well‐being within healthcare service delivery systems.

At the professional level, the findings highlight an important responsibility for occupational health practitioners, hospital administrators, and clinical managers to recognize hospital cleaning work as a high‐risk psychosocial occupation. Professional practice in nursing, environmental health, and occupational medicine is expected to incorporate regular screening for stress‐related symptoms, early identification of fatigue‐related functional impairment, and interdisciplinary coordination to address both physical and psychological dimensions of worker health. This reinforces the need for stronger occupational health integration within hospital management systems.

At the programmatic level, hospital occupational health programs require expansion beyond infection control and injury prevention to include structured stress monitoring, workload assessment, and psychosocial support mechanisms. Routine workplace risk assessment systems need to incorporate indicators such as dizziness, respiratory distress, and sprain and strain discomfort as early warning signs of excessive occupational strain. Continuous training and supervisory systems within hospital settings become critical for early detection and mitigation of stress‐related health outcomes. At the individual level, hospital cleaners experience direct physiological and psychological consequences of sustained occupational stress, as reflected in symptoms such as muscular discomfort, perspiration, dizziness, and breathing difficulty. These manifestations indicate reduced physical resilience and potential decline in work capacity over time. Individual coping capacity is influenced by working conditions, task intensity, and environmental exposures, emphasizing the importance of supportive work environments that reduce strain and enable recovery during and after shifts.

### 5.5 Limitations and Strengths of the Study

#### 5.5.1 Limitations of the Study

Despite its significant contributions, the present study has several limitations that should be considered in future research. First, the study relied entirely on self‐reported information from hospital cleaners in selected public hospitals over a 12‐month period. This approach may introduce recall bias or memory‐related inaccuracies, potentially affecting the reliability of the findings. In addition, the absence of clinical records and administrative data from regional labor and health bureaus limited the ability to triangulate results and strengthen the evidence base.

Second, dichotomous (Yes/No) response options were used to quantify specific research variables. The purpose of these response alternatives was to make administration easier, enhance participants' comprehension, and enable uniform responses from employees with varying educational and professional backgrounds. Dichotomization, however, might have lessened response variability and sensitivity and might not have adequately represented the frequency or intensity of some experiences. This should be taken into account when evaluating the results since it may have reduced the validity and precision of the measured constructs. Third, the study was confined to hospital cleaners working in selected public hospitals. As a result, the findings may not be generalizable to other occupational groups or fully capture the broader range of occupational health outcomes experienced by hospital cleaners in different settings.

Fourth, some participants may have been reluctant to provide honest responses due to concerns that their answers could negatively affect the reputation or performance of their hospitals and collaborating institutions. Such response bias may have influenced the accuracy of the reported data and could also affect future workplace interventions and decision‐making related to occupational stress management. Finally, the cross‐sectional design of the study limits the ability to establish causal relationships between variables. Therefore, future research should consider longitudinal study designs to better examine temporal relationships and causality.

#### 5.5.2 Strengths of the Study

Despite certain limitations, the present study has several notable strengths. First, it employed a quantitative research design, which is considered to generate robust and objective evidence that can inform policymakers in developing appropriate interventions. Second, the study utilized standardized instruments and validated measures derived from evidence across high‐, middle‐, and low‐income countries, ensuring balanced and comparable findings on job stress among hospital cleaners. Third, the research was conducted across three regional states and one administrative city, encompassing populations with diverse demographic characteristics. This broad coverage enhances the generalizability of the findings for occupational outcomes among sanitation workers in public hospitals both nationally and in other low‐ and middle‐income settings. Fourth, unlike many previous studies, this investigation used a relatively large and robust sample size (*n* = 741), which increases the reliability and statistical power of the results. Finally, the study applied appropriate and flexible multivariable statistical models to identify predictors while minimizing confounding effects, thereby strengthening the validity of the observed associations and supporting more accurate interpretation of determinants.

## 6 Conclusion

The current study concludes that approximately one‐third of hospital cleaners reported experiencing occupational stress in their workplace. These findings highlighted that a substantial proportion of cleaners are exposed to psychosocial and physical burdens during their routine duties in hospital settings. The reported level of stress reflects the demanding nature of cleaning tasks in healthcare environments, where exposure to infectious materials, time pressure, and insufficient support may contribute to elevated stress levels among workers.

The study also concludes that people reported experiencing symptoms of WRS such as headaches, low back pain, respiratory discomfort, dyspepsia, muscle soreness, heavy sweating, disorientation, and difficulty sleeping. These results suggest that hospital cleaners have physical and psychological symptoms as a result of occupational stress, which may have an impact on day‐to‐day functioning and productivity. Inadequate training, inadequate supervision, longer workdays, cigarette smoking, and chewing khat were all strongly associated with WRS. It also found that the level of WRS was also raised by sleep difficulties, unfavorable working conditions, job dissatisfaction, and little social acknowledgment for the cleaners at work setting. Therefore, in order to improve hospital cleaners' well‐being, productivity, and retention, complete interventions are required, including better working conditions, more social and supervisory support, sufficient training, and workplace health promotion methods.

## Author Contributions

**Sina Temesgen Tolera:** conceptualization, investigation, writing – original draft, visualization, writing – review and editing, formal analysis, software, data curation, supervision, validation, methodology. **Dawit Kassaye Getaneh:** conceptualization, writing – original draft, writing – review and editing, formal analysis. **Ararso Tafesse:** writing – original draft, writing – review and editing, data curation. **Fethia Mohammed:** writing – review and editing, writing – original draft. **Roba Argaw Tesema:** methodology, writing – review and editing, writing – original draft.

## Funding

The authors have nothing to report.

## Disclosure

The corresponding author affirms that this manuscript is an honest, accurate, and transparent account of the study being reported. The author had full access to all of the data in this study and takes complete responsibility for the integrity of the data and the accuracy of the data analysis. All important aspects of the study have been fully described, and no essential information has been omitted. Any deviations or discrepancies from the original study protocol have been clearly acknowledged and appropriately explained within the manuscript. The authors confirm that the work has been conducted and reported in accordance with accepted ethical and scientific standards.

## Conflicts of Interest

The authors declare no conflicts of interest.

## Supporting information


Supporting File 1

Supporting File 2

## Data Availability

The data that support the findings of this study are available from the corresponding author upon reasonable request.
