# Supplementary material for: Work‐Related Stress Among Neglected Work Forces in Tertiary Hospitals, Eastern Ethiopia: A Cross‐Sectional Study
Source: Health Sci Rep. 2026 Oct 10;9(10):e73374. doi: 10.1002/hsr2.73374 (PMC13653264; doi:10.1002/hsr2.73374)
Supplement: Supplementary file 1 [file HSR2-9-e73374-s002.pdf]

Information Sheet and Informed Voluntary Consent Form for Tertiary Hospitals in Eastern Ethiopia (English Language version)

Introduction

My name is Sina Temesgen Tolera. I am the Principal Investigator of the study entitled "Work-Related Stress among Neglected Work Forces in Tertiary Hospitals, Eastern Ethiopia: A Cross-Sectional Study," being conducted at Haramaya University, College of Health and Medical Sciences. I kindly request you to lend me your attention so that I can explain the purpose of the study, the procedures involved, and the participation of employees in this research.

The Study Title

Work-Related Stress among Neglected Work Forces in Tertiary Hospitals, Eastern Ethiopia: A Cross-Sectional Study

Purpose of the Study

The main purpose of this study is to assess the magnitude of work-related stress and identify factors associated with work-related stress among neglected work forces working in tertiary hospitals in Eastern Ethiopia. The study is also conducted as part of academic and research activities at Haramaya University. The findings will provide evidence that may help hospital administrators, health-sector managers, and other stakeholders understand workplace stressors and develop appropriate strategies to improve employees' working conditions, well-being, and productivity.

Procedure and Duration

Data collectors will administer a structured questionnaire to eligible employees working in selected tertiary hospitals. The questionnaire will contain questions related to socio-demographic characteristics, working conditions, occupational factors, workplace experiences, and work-related stress. Completing the questionnaire is expected to take approximately 30–40 minutes. Therefore, we kindly request your institution to allow the study to be conducted among eligible employees.

Risks and Benefits

There are no anticipated major physical risks associated with participation in this study. However, some questions concerning workplace experiences or stress may cause minor psychological discomfort or inconvenience. Participants may decline to answer any question they

do not wish to answer. There will be no direct financial payment or other direct reward for participating in the study.

Although participants may not receive a direct benefit, the findings may provide important information for tertiary hospitals, health managers, policymakers, and other stakeholders to identify workplace challenges and develop interventions to reduce work-related stress and improve working environments.

Confidentiality

All information collected during this study will be kept strictly confidential. No participant's name or other personally identifying information will be included in the research report. Participants will be identified using codes rather than names. The information obtained will be used only for research purposes, and findings will be presented in aggregate form so that individual participants cannot be identified.

Rights

Permission to allow this study to be conducted is entirely voluntary. You have the right to permit or refuse the study on behalf of your institution. If you permit the study, you have the right to request that the study be stopped within your institution if you observe any misleading, inappropriate, or unethical procedures during the data collection process. Refusal to permit the study will not result in any penalty or loss of institutional benefits.

Research Team

- Sina Temesgen Tolera – Principal Investigator
- Roba Argaw Tesema – Research Team Member
- Dawit Kassaye Getaneh – Research Team Member
- Ararso Tafesse – Research Team Member

Principal Investigator's Contact Address

If you have any questions or inquiries about the study or its procedures, please contact:

Sina Temesgen Tolera

Principal Investigator

Mobile: +251 913 023 634

Email: sinetem3@gmail.com

IHRERC Contact Address

Institutional Health Research Ethics Review Committee (IHRERC)
Haramaya University, College of Health and Medical Sciences
Office Phone: +251-254-66-2012
P.O. Box 235, Harar, Ethiopia

Declaration of Informed Voluntary Consent

I have read and understood the information provided in this information sheet. I have been clearly informed about the purpose of the study, procedures, possible risks and benefits, confidentiality, voluntary participation, and contact addresses for further inquiries. I have been given the opportunity to ask questions and received satisfactory answers to my questions.

I understand that participation is voluntary and that I have the right to refuse or withdraw permission at any time if I observe misleading, inappropriate, or unethical procedures during the data collection process. Therefore, I voluntarily give permission on behalf of ______________________________ Tertiary Hospital for this study to be conducted among eligible employees in the institution.

Name and signature of Hospital Representative/Head:

Name: ______________________________

Signature: ___________________________

Date: _______________________________

Name and signature of Principal Investigator:

Name: Sina Temesgen Tolera

Signature: ___________________________

Date: _______________________________

Informed Voluntary Consent Form for Study Participants (>18 Years) – English Version

Introduction

My name is ______________________________. I am working as a data collector for the study entitled "Work-Related Stress among Neglected Work Forces in Tertiary Hospitals, Eastern Ethiopia: A Cross-Sectional Study," being conducted by Sina Temesgen Tolera and the research team at Haramaya University, College of Health and Medical Sciences. I kindly request you to lend me your attention so that I can explain the study and your participation in it.

The Study Title

Work-Related Stress among Neglected Work Forces in Tertiary Hospitals, Eastern Ethiopia: A Cross-Sectional Study

Purpose of the Study

The main purpose of this study is to assess the magnitude of work-related stress and identify factors associated with work-related stress among neglected work forces working in tertiary hospitals in Eastern Ethiopia. The findings will provide evidence that may help hospital administrators, health managers, and other stakeholders identify workplace challenges and develop appropriate strategies to improve working conditions, employee well-being, and the quality of services provided.

Procedure and Duration

If you agree to participate, I will administer a structured questionnaire to you. The questions will cover your socio-demographic characteristics, working conditions, occupational factors, workplace experiences, and work-related stress. The questionnaire is expected to take approximately 30–40 minutes to complete. I kindly request you to spare this amount of time to participate in the study.

Risks and Benefits

There are no anticipated major physical risks from participating in this study. However, some questions about workplace experiences or stress may cause minor discomfort. You have the right to skip any question that you do not wish to answer.

There will be no direct financial payment or reward for participating in this study. However, the findings may provide useful information to your hospital and other stakeholders for understanding workplace stress and developing measures to improve working conditions and employee well-being.

Confidentiality

All information collected from you will be kept strictly confidential. Your name will not be recorded in the research report, and no information that directly identifies you will be disclosed.

A unique code may be used instead of your name. The information you provide will be used only for research purposes, and the findings will be reported in aggregate form.

Rights

Participation in this study is completely voluntary. You have the right to decide whether or not to participate. If you agree to participate, you may withdraw from the study at any time without giving a reason. You may also refuse to answer any question that you do not want to answer. Your decision to participate, refuse, or withdraw will not affect your employment, rights, benefits, or relationship with your institution.

Research Team

- Sina Temesgen Tolera – Principal Investigator
- Roba Argaw Tesema – Research Team Member
- Dawit Kassaye Getaneh – Research Team Member
- Ararso Tafesse – Research Team Member

Principal Investigator's Contact Address

If you have any questions or inquiries about the study or its procedures, please contact:

Sina Temesgen Tolera
Principal Investigator
Mobile: +251 913 023 634
Email: sinetem3@gmail.com

IHRERC Contact Address

Institutional Health Research Ethics Review Committee (IHRERC)
Haramaya University, College of Health and Medical Sciences
Office Phone: +251-254-66-2012
P.O. Box 235, Harar, Ethiopia

Declaration of Informed Voluntary Consent

I have read and understood the information provided in this information sheet. I have been informed about the purpose of the study, procedures, possible risks and benefits, confidentiality, voluntary participation, and my rights as a participant. I have been given the opportunity to ask questions and have received satisfactory answers to any questions that were unclear to me.

I understand that my participation is completely voluntary. I understand that I can refuse to answer any question or withdraw from the study at any time without losing any rights or benefits to which I am otherwise entitled.

Therefore, I voluntarily agree to participate in the study entitled “Work-Related Stress among Neglected Work Forces in Tertiary Hospitals, Eastern Ethiopia: A Cross-Sectional Study,” as indicated by my signature below.

Name and signature of Study Participant:
Name: ______________________________
Signature: ___________________________
Date: _______________________________

Name and signature of Data Collector:
Name: ______________________________
Signature: ___________________________
Date: _______________________________

7. 3 Questionnaire: Work-Related Stress Among Hospital Cleaners in Eastern Ethiopia

Introduction to the participant

Dear participant,
This questionnaire is designed to assess work-related stress, working conditions, behavioral factors, and environmental factors among hospital cleaners. Your responses will be kept confidential and used only for research purposes. There are no right or wrong answers. Please answer each question honestly based on your experience.

Instruction: For most questions, please tick (✓) the response that best describes your situation.

Section I. Socio-Demographic and Work-Related Characteristics

1. Sex

What is your sex?

☐ 1. Female
☐ 2. Male

2. Age

How old are you? _______ years

For analysis:

☐ 1. Less than 24 years
☐ 2. 25–35 years
☐ 3. Greater than 35 years

3. Marital status

☐ 1. Single
☐ 2. Married
☐ 3. Separated/divorced

4. Highest educational level completed

☐ 1. Below Grade 5
☐ 2. Grade 5–8
☐ 3. Above Grade 8

5. Total work experience as a hospital cleaner

How many years have you worked as a cleaner?

______ years

For analysis:

☐ 1. Less than 2 years
☐ 2. 3–5 years
☐ 3. Greater than 5 years

6. Type of employment

☐ 1. Permanent
☐ 2. Contractual

7. Monthly salary

What is your approximate monthly salary in US dollars?

$ _________

For analysis:

☐ 1. Less than $21
☐ 2. $21–44
☐ 3. Greater than $44

If data are collected in Ethiopian Birr, record the actual salary in ETB and convert to USD using the exchange rate specified in the study protocol.

8. Number of work shifts per day

☐ 1. One shift
☐ 2. Two shifts
☐ 3. Three shifts

Section II. Workplace Supervision and Occupational Health and Safety

9. How frequently are you supervised by your immediate supervisor regarding your cleaning work?

☐ 1. No supervision
☐ 2. Sometimes
☐ 3. Regularly

10. Have you ever received occupational health and safety (OHS) training related to your cleaning work?

☐ 1. Yes
☐ 2. No

11. If yes, when did you most recently receive OHS training?

☐ 1. Within the last 6 months
☐ 2. 7–12 months ago
☐ 3. More than 1 year ago
☐ 4. Don't remember

12. Does your workplace provide you with adequate personal protective equipment (PPE)?

☐ 1. Yes
☐ 2. No

13. Are you able to obtain the PPE you need whenever you require it?

☐ 1. Always
☐ 2. Sometimes
☐ 3. Never

Section III. Workload and Working Hours

14. Do you consider your current workload to be heavy?

☐ 1. Yes
☐ 2. No

15. On average, how many hours do you work per day?

______ hours/day

16. Do you usually work more than 8 hours per day?

☐ 1. Yes
☐ 2. No

17. How often are you required to work beyond your scheduled working hours?

☐ 1. Never
☐ 2. Rarely
☐ 3. Sometimes
☐ 4. Often
☐ 5. Always

18. Do you have sufficient time to complete your assigned cleaning tasks?

☐ 1. Yes
☐ 2. No

19. Do you feel that the number of cleaning tasks assigned to you is too high?

☐ 1. Yes
☐ 2. No

Section IV. Work-Related Stress

20. During the last one month, have you experienced work-related stress because of your job as a hospital cleaner?

☐ 1. Yes
☐ 2. No

Coding:
Yes = 1
No = 0

21. If yes, what were the main sources of your work-related stress?

Multiple responses are allowed.

☐ 1. Heavy workload
☐ 2. Long working hours
☐ 3. Inadequate staffing
☐ 4. Poor supervision
☐ 5. Poor recognition of my work
☐ 6. Low salary
☐ 7. Poor working environment
☐ 8. Lack of PPE
☐ 9. Exposure to hazardous materials
☐ 10. Conflict with coworkers
☐ 11. Conflict with supervisors
☐ 12. Other: ____________

Section V. Stress-Related Physical and Psychological Symptoms

Instruction: If you answered Yes to Question 20, please indicate how frequently/severely you experienced each symptom during the previous one month.

No.	Symptom	None	Mild	Moderate	Severe
22	Body shaking/trembling	☐	☐	☐	☐
23	Dizziness	☐	☐	☐	☐
24	Excessive perspiration/sweating	☐	☐	☐	☐
25	Muscle soreness	☐	☐	☐	☐
26	Headache	☐	☐	☐	☐
27	Backache	☐	☐	☐	☐
28	Disturbed sleep	☐	☐	☐	☐
29	Difficulty breathing/respiratory discomfort	☐	☐	☐	☐
30	Indigestion/stomach discomfort	☐	☐	☐	☐
31	Feeling tired or exhausted	☐	☐	☐	☐
32	Irritability	☐	☐	☐	☐
33	Difficulty concentrating	☐	☐	☐	☐

Suggested coding:
0 = None
1 = Mild
2 = Moderate
3 = Severe

Section VI. Sleep

34. During the past month, have you experienced sleeping problems/disorders?

☐ 1. Yes
☐ 2. No

35. If yes, what type of sleeping problem did you experience?

☐ 1. Difficulty falling asleep
☐ 2. Waking frequently during the night
☐ 3. Waking too early

☐ 4. Poor-quality sleep
☐ 5. Other: ____________

36. On average, how many hours do you sleep per night?

_____ hours

Section VII. Alcohol Consumption

37. Do you currently consume alcoholic beverages?

☐ 1. Yes
☐ 2. No

38. During the past 30 days, how often did you consume alcohol?

☐ 1. Never
☐ 2. Less than once per week
☐ 3. 1–2 days per week
☐ 4. 3–4 days per week
☐ 5. 5 or more days per week

39. When drinking alcohol, would you describe your consumption as heavy?

☐ 1. Yes
☐ 2. No

For the main analysis:
Heavy alcohol consumption: Yes/No.

Section VIII. Khat/Cath Use

40. Do you currently chew khat (chat)?

☐ 1. Yes
☐ 2. No

41. During the past 30 days, how frequently did you chew khat?

☐ 1. Never
☐ 2. Less than once per week
☐ 3. 1–2 days per week
☐ 4. 3–4 days per week
☐ 5. 5 or more days per week

42. How many hours do you usually spend chewing khat on a chewing day?

______ hours

Section IX. Cigarette Smoking

43. Do you currently smoke cigarettes?

☐ 1. Yes
☐ 2. No

44. Have you smoked cigarettes regularly in the past?

☐ 1. Yes
☐ 2. No

45. If currently smoking, approximately how many cigarettes do you smoke per day?

______ cigarettes

Section X. Job Satisfaction

46. Overall, are you satisfied with your current job as a hospital cleaner?

☐ 1. Yes
☐ 2. No

47. How satisfied are you with your salary?

☐ 1. Very satisfied
☐ 2. Satisfied
☐ 3. Neutral
☐ 4. Dissatisfied
☐ 5. Very dissatisfied

48. How satisfied are you with your working hours?

☐ 1. Very satisfied
☐ 2. Satisfied
☐ 3. Neutral
☐ 4. Dissatisfied
☐ 5. Very dissatisfied

49. How satisfied are you with your relationship with your supervisor?

☐ 1. Very satisfied
☐ 2. Satisfied
☐ 3. Neutral
☐ 4. Dissatisfied
☐ 5. Very dissatisfied

50. How satisfied are you with your relationship with other workers?

☐ 1. Very satisfied
☐ 2. Satisfied
☐ 3. Neutral
☐ 4. Dissatisfied
☐ 5. Very dissatisfied

Section XI. Environmental Satisfaction

51. Overall, are you satisfied with your working environment?

☐ 1. Yes
☐ 2. No

52. Are you satisfied with the cleanliness and safety of your workplace?

☐ 1. Yes
☐ 2. No

53. Are you satisfied with the availability of cleaning materials and equipment?

☐ 1. Yes
☐ 2. No

54. Are you satisfied with ventilation in your workplace?

☐ 1. Yes
☐ 2. No

55. Are you satisfied with lighting in your workplace?

☐ 1. Yes
☐ 2. No

56. Are you satisfied with the availability of water and sanitation facilities?

☐ 1. Yes

☐ 2. No

Section XII. Recognition and Social Support

57. Do you feel that your work as a hospital cleaner is adequately recognized by hospital management?

☐ 1. Yes

☐ 2. No

58. Do you feel that hospital staff respect the work you perform?

☐ 1. Yes

☐ 2. No

59. Do you receive appreciation when you perform your work well?

☐ 1. Yes

☐ 2. No

60. Do you feel that your contribution to patient safety and hospital infection prevention is recognized?

☐ 1. Yes

☐ 2. No

61. Do you experience poor social recognition of your work?

☐ 1. Yes

☐ 2. No

Section XIII. Additional Workplace Factors

62. Do you have adequate cleaning equipment to perform your duties?

☐ 1. Yes

☐ 2. No

63. Do you have adequate cleaning supplies/materials?

☐ 1. Yes

☐ 2. No

64. Do you frequently perform physically demanding cleaning activities?

☐ 1. Yes

☐ 2. No

65. Are you exposed to unpleasant odors during your work?

☐ 1. Yes

☐ 2. No

66. Are you exposed to chemical cleaning agents?

☐ 1. Yes

☐ 2. No

67. Do you feel safe while performing your cleaning duties?

☐ 1. Yes

☐ 2. No

68. Do you receive adequate support from your immediate supervisor when you have work-related problems?

☐ 1. Always

☐ 2. Sometimes

☐ 3. Never

Thank you in advance for participating on this study

7.1. በምሥራቅ ኢትዮጵያ ባሉ ሦስተኛ ደረጃ ሆስፒታሎች ለሚካሄድ ጥናት የመረጃ ማብራሪያ ቅጽና የተሳትፎ በፈቃደኝነት ስምምነት ቅጽ(የአማርኛ ቋንቋ ስሪት)

መግቢያ

ስሜ ሲና ተመስገን ቶለራ ነው። በሐረማያ ዩኒቨርሲቲ፣ የጤናና ሕክምና ሳይንስ ኮሌጅ በሚካሄደው "በምሥራቅ ኢትዮጵያ ባሉ ሦስተኛ ደረጃ ሆስፒታሎች በተለይ ችላ ተብለው በሚታዩ የሥራ ኃይሎች ላይ ከሥራ ጋር የተያያዘ ጭንቀት፡ የክሮስ-ሴክሽናል ጥናት" የተሰኘው ጥናት ዋና ተመራማሪ ነኝ። የጥናቱን ዓላማ፣ የሚከናወኑ ሂደቶችንና የሠራተኞችን ተሳትፎ በተመለከተ ማብራሪያ እንድሰጥዎ እባክዎ ትኩረትዎን እንዲሰጡኝ እጠይቃለሁ።

የጥናቱ ርዕስ

በምሥራቅ ኢትዮጵያ ባሉ ሦስተኛ ደረጃ ሆስፒታሎች በተለይ ችላ ተብለው በሚታዩ የሥራ ኃይሎች ላይ ከሥራ ጋር የተያያዘ ጭንቀት፡ የክሮስ-ሴክሽናል ጥናት

የጥናቱ ዓላማ

የዚህ ጥናት ዋና ዓላማ በምሥራቅ ኢትዮጵያ ባሉ ሦስተኛ ደረጃ ሆስፒታሎች ውስጥ በሚሠሩ ችላ ተብለው በሚታዩ የሥራ ኃይሎች መካከል ከሥራ ጋር የተያያዘ ጭንቀት መጠንን መለካትና ከሥራ ጋር የተያያዘ ጭንቀት ጋር የተያያዙ ምክንያቶችን መለየት ነው። ጥናቱ በተጨማሪም በሐረማያ ዩኒቨርሲቲ የአካዳሚክና የምርምር ሥራ አካል ሆኖ ይከናወናል። የጥናቱ ውጤት የሆስፒታል አስተዳደሮች፣ የጤና ዘርፍ አመራሮችና ሌሎች ባለድርሻ አካላት የሥራ ቦታ ጭንቀትን የሚያስከትሉ ምክንያቶችን እንዲረዱና የሠራተኞችን የሥራ ሁኔታ፣ ደህንነትና ምርታማነት ለማሻሻል ተገቢ ስልቶችን እንዲያዘጋጁ የሚያግዝ ማስረጃ ይሰጣል።

የጥናቱ ሂደትና የሚፈጀው ጊዜ

የመረጃ ሰብሳቢዎች በተመረጡ ሦስተኛ ደረጃ ሆስፒታሎች ውስጥ ለሚሠሩ ብቁ ሠራተኞች የተዘጋጀ መዋቅራዊ መጠይቅ ያቀርባሉ። መጠይቁ ስለ ማኅበራዊና ሥነ-ሕዝብ ባህሪያት፣ የሥራ ሁኔታዎች፣ የሙያ ምክንያቶች፣ የሥራ ቦታ ልምዶችና ከሥራ ጋር የተያያዘ ጭንቀት ጥያቄዎችን ይይዛል። መጠይቁን ለመሙላት በግምት 30–40 ደቂቃ ይወስዳል። ስለዚህ ጥናቱ በብቁ ሠራተኞች መካከል እንዲካሄድ ተቋማችሁ ፈቃድ እንዲሰጥ በአክብሮት እንጠይቃለን።

ሊኖሩ የሚችሉ አደጋዎችና ጥቅሞች

በዚህ ጥናት በመሳተፍ ምክንያት ከፍተኛ የአካል ጉዳት ወይም አደጋ እንደሚደርስ አይጠበቅም። ሆኖም ከሥራ ቦታ ልምድ ወይም ከጭንቀት ጋር የተያያዙ አንዳንድ ጥያቄዎች ትንሽ የሥነ-ልቦና ምቾት ማጣት ወይም አለመመቸት ሊያስከትሉ ይችላሉ። ተሳታፊዎች መመለስ የማይፈልጉትን ማንኛውንም ጥያቄ መተው ይችላሉ። በጥናቱ ለመሳተፍ ቀጥተኛ የገንዘብ ክፍያ ወይም ሌላ ቀጥተኛ ሽልማት አይኖርም።

ተሳታፊዎች ቀጥተኛ ጥቅም ላያገኙ ቢችሉም፣ የጥናቱ ውጤት ለሦስተኛ ደረጃ ሆስፒታሎች፣ ለጤና አመራሮች፣ ለፖሊሲ አውጪዎችና ለሌሎች ባለድርሻ አካላት የሥራ ቦታ ችግሮችን ለመለየት፣ ከሥራ ጋር የተያያዘ ጭንቀትን ለመቀነስና የሥራ አካባቢን ለማሻሻል ጠቃሚ መረጃ ሊሰጥ ይችላል።

ምስጢራዊነት

በዚህ ጥናት ወቅት የሚሰበሰብ ማንኛውም መረጃ በጥብቅ ምስጢራዊ ይደረጋል። የማንኛውም ተሳታፊ ስም ወይም ሌላ በቀጥታ ለመለየት የሚያስችል መረጃ በጥናቱ ሪፖርት ውስጥ አይካተትም። ተሳታፊዎች በስም ምትክ በኮድ ይለያሉ። የሚሰበሰበው መረጃ ለምርምር ዓላማ ብቻ የሚውል ሲሆን፣ ውጤቱም የግለሰብ ተሳታፊዎችን ለመለየት በማይቻል የጥቅል መልኩ ይቀርባል።

መብቶች

ይህ ጥናት በተቋማችሁ እንዲካሄድ ፈቃድ መስጠት ሙሉ በሙሉ በፈቃደኝነት የሚወሰን ነው። ተቋማችሁን ወክለው ጥናቱን ለመፍቀድ ወይም ላለመፍቀድ መብት አላችሁ። ጥናቱን ከፈቀዳችሁ በኋላ በመረጃ ሰብሳቢዎች ሂደት ወቅት አሳሳች፣ ተገቢ ያልሆነ ወይም ከሥነ-ምግባር ውጪ የሆነ አሠራር ካስተዋላችሁ በተቋማችሁ ውስጥ ጥናቱ እንዲቆም መጠየቅ ትችላላችሁ። ጥናቱን ላለመፍቀድ ምንም ዓይነት ቅጣት ወይም የተቋማዊ ጥቅም ማጣት አያስከትልም።

የምርምር ቡድን

- ሲና ተመስገን ቶለራ – ዋና ተመራማሪ
- ሮባ አርጋው ተሰማ – የምርምር ቡድን አባል
- ዳዊት ካሳዬ ጌታነህ – የምርምር ቡድን አባል
- አራርሶ ታፈሰ – የምርምር ቡድን አባል

የዋና ተመራማሪ የመገኛ አድራሻ

ስለ ጥናቱ ወይም ስለ ጥናቱ ሂደት ማንኛውም ጥያቄ ካለዎት፦

ሲና ተመስገን ቶለራ
ዋና ተመራማሪ
ስልክ፦ +251 913 023 634
ኢሜይል፦ sinetem3@gmail.com

የIHRERC የመገኛ አድራሻ

Institutional Health Research Ethics Review Committee (IHRERC)
ሐረማያ ዩኒቨርሲቲ፣ የጤናና ሕክምና ሳይንስ ኮሌጅ
የቢሮ ስልክ፦ +251-254-66-2012
ፖ.ሳ.ቁ፦ 235፣ ሐረር፣ ኢትዮጵያ

የተረዳሁበትና በፈቃደኝነት የሰጠሁት ስምምነት መግለጫ

በዚህ የመረጃ ማብራሪያ ቅጽ የተሰጠውን መረጃ አንብቤ ተረድቻለሁ። ስለ ጥናቱ ዓላማ፣ ሂደት፣ ሊኖሩ ስለሚችሉ አደጋዎችና ጥቅሞች፣ ምስጢራዊነት፣ በፈቃደኝነት ስለሚደረግ ተሳትፎና ተጨማሪ መረጃ ለማግኘት ስለሚቻልባቸው የመገኛ አድራሻዎች በግልጽ መረጃ ተሰጥቶኛል። ጥያቄ ለመጠየቅ ዕድል ተሰጥቶኝ ለጥያቄዎቼም አጥጋቢ መልስ አግኝቻለሁ።

ተሳትፎው በፈቃደኝነት የሚደረግ መሆኑን እና በመረጃ ሰብሳቢዎች ሂደት ወቅት አሳሳች፣ ተገቢ ያልሆነ ወይም ከሥነ-ምግባር ውጪ የሆነ አሠራር ካስተዋልኩ ፈቃዴን በማንኛውም ጊዜ ማንሳት ወይም ጥናቱ እንዲቆም መጠየቅ እንደምችል ተረድቻለሁ። ስለዚህ፣ ________________________________ ሦስተኛ ደረጃ ሆስፒታል በሚገኙ ብቁ ሠራተኞች መካከል ይህ ጥናት እንዲካሄድ በፈቃዴ እፈቅዳለሁ።

የሆስፒታል ተወካይ/ኃላፊ ስምና ፊርማ፡-

ስም፡- ________________________________

ፊርማ፡- ______________________________

ቀን፡- ________________________________

የዋና ተመራማሪ ስምና ፊርማ፡-

ስም፡- ሲና ተመስገን ቶለራ

ፊርማ፡- ______________________________

ቀን፡- ________________________________

7.2. ከ18 ዓመት በላይ ለሆኑ የጥናቱ ተሳታፊዎች የተረዳሁበትና በፈቃደኝነት የተሰትፎ ስምምነት ቅጽ(የአማርኛ ቋንቋ ስሪት)

መግቢያ

ስሜ ________________________________ ነው። በሐረማያ ዩኒቨርሲቲ፣ የጤናና ሕክምና ሳይንስ ኮሌጅ በሲና ተመስገን ቶለራ እና በምርምር ቡድኑ እየተካሄደ ላለው "በምሥራቅ ኢትዮጵያ ባሉ ሦስተኛ ደረጃ ሆስፒታሎች በተለይ ችላ ተብለው በሚታዩ የሥራ ኃይሎች ላይ ከሥራ ጋር የተያያዘ ጭንቀት፡ የክሮስ-ሴክሽናል ጥናት" የተሰኘው ጥናት የመረጃ ሰብሳቢ ነኝ። ስለ ጥናቱና በጥናቱ ስለሚኖረዎት ተሳትፎ ማብራሪያ እንድሰጥዎ እባክዎ ትኩረትዎን እንዲሰጡኝ እጠይቃለሁ።

የጥናቱ ርዕስ

በምሥራቅ ኢትዮጵያ ባሉ ሦስተኛ ደረጃ ሆስፒታሎች በተለይ ችላ ተብለው በሚታዩ የሥራ ኃይሎች ላይ ከሥራ ጋር የተያያዘ ጭንቀት፡ የክሮስ-ሴክሽናል ጥናት

የጥናቱ ዓላማ

የዚህ ጥናት ዋና ዓላማ በምሥራቅ ኢትዮጵያ ባሉ ሦስተኛ ደረጃ ሆስፒታሎች ውስጥ በሚሠሩ ችላ ተብለው በሚታዩ የሥራ ኃይሎች መካከል ከሥራ ጋር የተያያዘ ጭንቀት መጠንን መለካትና ከጭንቀቱ ጋር የተያያዙ ምክንያቶችን መለየት ነው። የጥናቱ ውጤት የሥራ ቦታ ችግሮችን ለመረዳትና የሥራ ሁኔታን፣ የሠራተኞችን ደህንነትና የሚሰጡ አገልግሎቶችን ጥራት ለማሻሻል ተገቢ ስልቶችን ለማዘጋጀት ለሆስፒታል አስተዳደሮች፣ ለጤና አመራሮችና ለሌሎች ባለድርሻ አካላት ማስረጃ ይሰጣል።

የጥናቱ ሂደትና የሚፈጀው ጊዜ

በጥናቱ ለመሳተፍ ከተስማሙ፣ የተዘጋጀ መዋቅራዊ መጠይቅ እንሰጥዎታለን። ጥያቄዎቹ ስለ ማኅበራዊና ሥነ-ሕዝብ ባህሪያት፣ የሥራ ሁኔታዎች፣ የሙያ ምክንያቶች፣ የሥራ ቦታ ልምዶችና ከሥራ ጋር የተያያዘ ጭንቀትን ይመለከታሉ። መጠይቁን ለመሙላት በግምት 30–40 ደቂቃ ይወስዳል።

አደጋዎችና ጥቅሞች

በጥናቱ በመሳተፍ ምክንያት ከፍተኛ የአካል አደጋ አይጠበቅም። ሆኖም ስለ ሥራ ቦታ ልምድ ወይም ጭንቀት የሚጠይቁ አንዳንድ ጥያቄዎች ትንሽ አለመመቸት ሊያስከትሉ ይችላሉ። መመለስ የማይፈልጉትን ማንኛውንም ጥያቄ መተው ይችላሉ።

በጥናቱ ለመሳተፍ ቀጥተኛ የገንዘብ ክፍያ ወይም ሽልማት አይኖርም። ሆኖም የጥናቱ ውጤት ለሆስፒታሉና ለሌሎች ባለድርሻ አካላት የሥራ ቦታ ጭንቀትን ለመረዳትና የሥራ ሁኔታንና የሠራተኞችን ደህንነት ለማሻሻል ጠቃሚ መረጃ ሊሰጥ ይችላል።

ምስጢራዊነት

ከእርስዎ የሚሰበሰበው መረጃ በጥብቅ ምስጢራዊ ይደረጋል። ስምዎ በምርምር ሪፖርት ውስጥ አይመዘገብም፣ በቀጥታ ሊለይዎት የሚችል መረጃም አይገለጽም። ከስምዎ ይልቅ ልዩ ኮድ ሊሰጥዎ ይችላል። የሚሰጡት መረጃ ለምርምር ዓላማ ብቻ ይውላል፣ ውጤቱም የግለሰብ ተሳታፊን ለመለየት በማይቻል የጥቅል መልኩ ይቀርባል።

መብቶች

በዚህ ጥናት መሳተፍ ሙሉ በሙሉ በፈቃደኝነት ነው። ለመሳተፍ ወይም ላለመሳተፍ መወሰን ይችላሉ። ለመሳተፍ ከተስማሙ በኋላም ምክንያት ሳይሰጡ በማንኛውም ጊዜ ከጥናቱ መውጣት ይችላሉ። መመለስ የማይፈልጉትን ጥያቄ መመለስ ላለመፈለግም መብት አለዎት። ለመሳተፍ፣ ላለመሳተፍ ወይም ከጥናቱ ለመውጣት የሚወስኑት ውሳኔ በሥራዎ፣ በመብቶችዎ፣ በጥቅሞችዎ ወይም ከተቋሙ ጋር ባለዎት ግንኙነት ላይ ምንም ተጽዕኖ አያሳድርም።

የምርምር ቡድን

- ሲና ተመስገን ቶለራ – ዋና ተመራማሪ
- ሮባ አርጋው ተሰማ – የምርምር ቡድን አባል
- ዳዊት ካሳዬ ጌታነህ – የምርምር ቡድን አባል
- አራርሶ ታፈሰ – የምርምር ቡድን አባል

የዋና ተመራማሪ የመገኛ አድራሻ

ሲና ተመስገን ቶለራ
ዋና ተመራማሪ
ስልክ፦ +251 913 023 634
ኢሜይል፦ sinetem3@gmail.com

የIHRERC የመገኛ አድራሻ

Institutional Health Research Ethics Review Committee (IHRERC)
ሐረማያ ዩኒቨርሲቲ፣ የጤናና ሕክምና ሳይንስ ኮሌጅ
የቢሮ ስልክ፦ +251-254-66-2012
ፖ.ሳ.ቁ፦ 235፣ ሐረር፣ ኢትዮጵያ

የተረዳሁበትና በፈቃደኝነት የሰጠሁት ስምምነት መግለጫ

በዚህ የመረጃ ማብራሪያ ቅጽ የተሰጠውን መረጃ አንብቤ ተረድቻለሁ። ስለ ጥናቱ ዓላማ፣ ሂደት፣ ሊኖሩ ስለሚችሉ አደጋዎችና ጥቅሞች፣ ምስጢራዊነት፣ በፈቃደኝነት ስለሚደረግ ተሳትፎና እንደ ተሳታፊ ስላሉኝ መብቶች ተገቢው ማብራሪያ ተሰጥቶኛል። ጥያቄ ለመጠየቅ ዕድል ተሰጥቶኝ ለጥያቄዎቼም አጥጋቢ መልስ አግኝቻለሁ።

በጥናቱ መሳተፍ ሙሉ በሙሉ በፈቃደኝነት እንደሆነ ተረድቻለሁ። ማንኛውንም ጥያቄ ላለመመለስ ወይም በማንኛውም ጊዜ ከጥናቱ ለመውጣት መብት እንዳለኝ ተረድቻለሁ፤ ይህም በሌሎች መብቶቼ ወይም ጥቅሞቼ ላይ ተጽዕኖ እንደማያሳድር ተረድቻለሁ።

ስለዚህ፣ "በምሥራቅ ኢትዮጵያ ባሉ ሦስተኛ ደረጃ ሆስፒታሎች በተለይ ችላ ተብለው በሚታዩ የሥራ ኃይሎች ላይ ከሥራ ጋር የተያያዘ ጭንቀት፡ የክሮስ-ሴክሽናል ጥናት" በተሰኘው ጥናት ለመሳተፍ በፈቃዴ ተስማምቻለሁ።

የጥናቱ ተሳታፊ ስምና ፊርማ፡-

ስም፡- ___________________________________

ፊርማ፡- ________________________________

ቀን፡- ____________________________________

የመረጃ ሰብሳቢ ስምና ፊርማ፡-

ስም፡- ___________________________________

ፊርማ፡- ________________________________

ቀን፡- ____________________________________

7.3. መጠይቅ፡- በምሥራቅ ኢትዮጵያ በሆስፒታል ጽዳት ሠራተኞች መካከል ከሥራ ጋር የተያያዘ ጭንቀት

ለተሳታፊው መግቢያ

ውድ ተሳታፊ፣

ይህ መጠይቅ በሆስፒታል ጽዳት ሠራተኞች መካከል ከሥራ ጋር የተያያዘ ጭንቀትን፣ የሥራ ሁኔታዎችን፣ የባህሪ ምክንያቶችንና የአካባቢ ሁኔታዎችን ለመገምገም የተዘጋጀ ነው። የሚሰጡት ምላሾች በምስጢር የሚጠበቁ ሲሆን ለምርምር ዓላማ ብቻ ይውላሉ። ትክክለኛ ወይም ስህተት የሆነ መልስ የለም። እባክዎ በራስዎ ልምድ መሠረት እያንዳንዱን ጥያቄ በታማኝነት ይመልሱ።

መመሪያ፡- በአብዛኛዎቹ ጥያቄዎች ሁኔታዎን በትክክል የሚገልጸውን መልስ (✓) ምልክት በማድረግ ይምረጡ።

ክፍል I. ማኅበራዊና ሥነ-ሕዝብ እና ከሥራ ጋር የተያያዙ ባህሪያት

1. ጾታ

ጾታዎ ምንድን ነው?

☐ 1. ሴት
☐ 2. ወንድ

2. ዕድሜ

ዕድሜዎ ስንት ነው? _______ ዓመት

ለትንተና፡-

☐ 1. ከ24 ዓመት በታች
☐ 2. 25–35 ዓመት
☐ 3. ከ35 ዓመት በላይ

3. የጋብቻ ሁኔታ

☐ 1. ያላገባ/ች
☐ 2. ያገባ/ች
☐ 3. ተለያይቷል/ታለች ወይም ፍቺ ያገኘ/ች

4. የተጠናቀቀ ከፍተኛ የትምህርት ደረጃ

☐ 1. ከ5ኛ ክፍል በታች
☐ 2. 5ኛ–8ኛ ክፍል
☐ 3. ከ8ኛ ክፍል በላይ

5. እንደ ሆስፒታል ጽዳት ሠራተኛ ያለዎት ጠቅላላ የሥራ ልምድ

እንደ ጽዳት ሠራተኛ ለስንት ዓመታት ሠርተዋል?
_____ ዓመት

ለትንተና፡-

☐ 1. ከ2 ዓመት በታች
☐ 2. 3–5 ዓመት
☐ 3. ከ5 ዓመት በላይ

6. የቅጥር ዓይነት

☐ 1. ቋሚ
☐ 2. ውል/ኮንትራት

7. ወርሃዊ ደመወዝ

በግምት ወርሃዊ ደመወዝዎ በአሜሪካ ዶላር ስንት ነው?

$ ________

ለትንተና፡-

☐ 1. ከ$21 በታች
☐ 2. $21–44
☐ 3. ከ$44 በላይ

መረጃው በኢትዮጵያ ብር ከተሰበሰበ፣ ትክክለኛውን የደመወዝ መጠን በETB ይመዝግቡ፤ ከዚያም በጥናቱ ፕሮቶኮል በተወሰነው የምንዛሬ ተመን ወደ USD ይቀይሩ።

8. በቀን የሚሠሩት የሥራ ፈረቃ ብዛት

☐ 1. አንድ ፈረቃ
☐ 2. ሁለት ፈረቃ
☐ 3. ሦስት ፈረቃ

ክፍል II. የሥራ ቦታ ቁጥጥርና የሙያ ጤናና ደህንነት

9. በጽዳት ሥራዎ ላይ በቅርብ የሚመለከትዎ ኃላፊ ምን ያህል ጊዜ ይቆጣጠርዎታል?

☐ 1. ምንም ቁጥጥር የለም
☐ 2. አንዳንድ ጊዜ
☐ 3. በመደበኛነት

10. ከጽዳት ሥራዎ ጋር በተያያዘ የሙያ ጤናና ደህንነት (OHS) ሥልጠና ወስደው ያውቃሉ?

☐ 1. አዎ

☐ 2. አይ

11. አዎ ካሉ፣ በቅርብ ጊዜ የOHS ሥልጠና የወሰዱት መቼ ነው?

☐ 1. ባለፉት 6 ወራት ውስጥ

☐ 2. ከ7–12 ወራት በፊት

☐ 3. ከ1 ዓመት በላይ በፊት

☐ 4. አላስታውስም

12. የሥራ ቦታዎ በቂ የግል መከላከያ መሣሪያ (PPE) ያቀርብልዎታል?

☐ 1. አዎ

☐ 2. አይ

13. በሚያስፈልግዎት ጊዜ የሚያስፈልግዎትን PPE ማግኘት ይችላሉ?

☐ 1. ሁልጊዜ

☐ 2. አንዳንድ ጊዜ

☐ 3. በፍጹም

ክፍል III. የሥራ ጫናና የሥራ ሰዓት

14. አሁን ያለዎት የሥራ ጫና ከባድ ነው ብለው ያስባሉ?

☐ 1. አዎ

☐ 2. አይ

15. በአማካይ በቀን ለስንት ሰዓት ይሠራሉ?

______ ሰዓት/ቀን

16. በተለምዶ በቀን ከ8 ሰዓት በላይ ይሠራሉ?

☐ 1. አዎ

☐ 2. አይ

17. ከተመደበልዎት የሥራ ሰዓት በላይ እንዲሠሩ ምን ያህል ጊዜ ይጠየቃሉ?

☐ 1. በፍጹም

☐ 2. አልፎ አልፎ

☐ 3. አንዳንድ ጊዜ

☐ 4. ብዙ ጊዜ

☐ 5. ሁልጊዜ

18. የተመደቡልዎትን የጽዳት ሥራዎች ለማጠናቀቅ በቂ ጊዜ አለዎት?

☐ 1. አዎ
☐ 2. አይ

19. የተመደቡልዎት የጽዳት ሥራዎች ብዛት ከመጠን በላይ ነው ብለው ይሰማዎታል?

☐ 1. አዎ
☐ 2. አይ

ክፍል IV. ከሥራ ጋር የተያያዘ ጭንቀት

20. ባለፈው አንድ ወር ውስጥ እንደ ሆስፒታል ጽዳት ሠራተኛ በሚሠሩት ሥራ ምክንያት ከሥራ ጋር የተያያዘ ጭንቀት አጋጥሞዎታል?

☐ 1. አዎ
☐ 2. አይ

ኮዲንግ፡-
አዎ = 1
አይ = 0

21. አዎ ካሉ፣ የከሥራ ጋር የተያያዘ ጭንቀትዎ ዋና ምንጮች ምንድን ናቸው?

ብዙ መልሶችን መምረጥ ይቻላል።

☐ 1. ከባድ የሥራ ጫና
☐ 2. ረጅም የሥራ ሰዓት
☐ 3. በቂ ያልሆነ የሠራተኛ ብዛት
☐ 4. ደካማ የሥራ ቁጥጥር
☐ 5. ለሥራዬ በቂ እውቅና አለመሰጠት
☐ 6. ዝቅተኛ ደመወዝ
☐ 7. ደካማ የሥራ አካባቢ
☐ 8. PPE አለመኖር
☐ 9. ለአደገኛ ነገሮች መጋለጥ
☐ 10. ከሥራ ባልደረቦች ጋር ግጭት
☐ 11. ከኃላፊዎች ጋር ግጭት
☐ 12. ሌላ፡- ____________

ክፍል V. ከጭንቀት ጋር የተያያዙ የአካልና የሥነ-ልቦና ምልክቶች

መመሪያ፡- በጥያቄ 20 “አዎ” ብለው ከመለሱ፣ ባለፈው አንድ ወር ውስጥ እያንዳንዱ ምልክት ምን ያህል ጊዜ/ክብደት እንደነበረው ይጠቁሙ።

ቁጥር	ምልክት	የለም	ቀላል	መካከለኛ	ከባድ
22	የሰውነት መንቀጥቀጥ	☐	☐	☐	☐
23	ማዞር	☐	☐	☐	☐
24	ከመጠን በላይ ላብ	☐	☐	☐	☐
25	የጡንቻ ሕመም	☐	☐	☐	☐
26	ራስ ምታት	☐	☐	☐	☐
27	የጀርባ ሕመም	☐	☐	☐	☐
28	የእንቅልፍ መዛባት	☐	☐	☐	☐
29	የመተንፈስ ችግር/የመተንፈሻ አለመመቸት	☐	☐	☐	☐
30	የምግብ መፍጫ ችግር/የሆድ አለመመቸት	☐	☐	☐	☐
31	ድካም ወይም ከፍተኛ መድከም	☐	☐	☐	☐
32	በቀላሉ መበሳጨት	☐	☐	☐	☐
33	ትኩረት ማድረግ መቸገር	☐	☐	☐	☐

የሚመከር ኮዲንግ፡-

0 = የለም
1 = ቀላል
2 = መካከለኛ
3 = ከባድ

ክፍል VI. እንቅልፍ

34. ባለፈው አንድ ወር ውስጥ የእንቅልፍ ችግር/መዛባት አጋጥሞዎታል?

☐ 1. አዎ
☐ 2. አይ

35. አዎ ካሉ፣ ምን ዓይነት የእንቅልፍ ችግር አጋጥሞዎታል?

☐ 1. እንቅልፍ ለመውሰድ መቸገር
☐ 2. በሌሊት በተደጋጋሚ መንቃት
☐ 3. በጣም ቀደም ብሎ መንቃት

☐ 4. ጥራት የሌለው እንቅልፍ
☐ 5. ሌላ፦ ____________

36. በአማካይ በአንድ ሌሊት ስንት ሰዓት ይተኛሉ?

_____ ሰዓት

ክፍል VII. የአልኮል መጠጥ አጠቃቀም

37. በአሁኑ ጊዜ የአልኮል መጠጥ ይጠጣሉ?

☐ 1. አዎ
☐ 2. አይ

38. ባለፉት 30 ቀናት ውስጥ አልኮል ምን ያህል ጊዜ ጠጥተዋል?

☐ 1. በፍጹም
☐ 2. በሳምንት ከአንድ ጊዜ በታች
☐ 3. በሳምንት 1–2 ቀን
☐ 4. በሳምንት 3–4 ቀን
☐ 5. በሳምንት 5 ቀን ወይም ከዚያ በላይ

39. አልኮል ሲጠጡ የሚጠጡትን መጠን ከፍተኛ/ብዙ ነው ብለው ይገልጹታል?

☐ 1. አዎ
☐ 2. አይ

ለዋናው ትንተና፦
ከፍተኛ የአልኮል አጠቃቀም፦ አዎ/አይ

ክፍል VIII. የጫት አጠቃቀም

40. በአሁኑ ጊዜ ጫት ይቅማሉ?

☐ 1. አዎ
☐ 2. አይ

41. ባለፉት 30 ቀናት ውስጥ ጫት ምን ያህል ጊዜ ቅመዋል?

☐ 1. በፍጹም
☐ 2. በሳምንት ከአንድ ጊዜ በታች
☐ 3. በሳምንት 1–2 ቀን
☐ 4. በሳምንት 3–4 ቀን
☐ 5. በሳምንት 5 ቀን ወይም ከዚያ በላይ

42. ጫት በሚቅሙበት ቀን በአማካይ ለስንት ሰዓት ጫት ይቅማሉ?

_____ ሰዓት

ክፍል IX. የሲጋራ ማጨስ

43. በአሁኑ ጊዜ ሲጋራ ያጨሳሉ?

☐ 1. አዎ
☐ 2. አይ

44. ቀደም ሲል በመደበኛነት ሲጋራ ያጨሱ ነበር?

☐ 1. አዎ
☐ 2. አይ

45. በአሁኑ ጊዜ ሲጋራ የሚያጨሱ ከሆነ፣ በቀን በግምት ስንት ሲጋራ ያጨሳሉ?

_____ ሲጋራ

ክፍል X. የሥራ እርካታ

46. በአጠቃላይ እንደ ሆስፒታል ጽዳት ሠራተኛ ባለዎት የአሁኑ ሥራ ደስተኛ/እርካታ አለዎት?

☐ 1. አዎ
☐ 2. አይ

47. በደመወዝዎ ምን ያህል እርካታ አለዎት?

☐ 1. በጣም እርካታ አለኝ
☐ 2. እርካታ አለኝ
☐ 3. መካከለኛ/ገለልተኛ
☐ 4. እርካታ የለኝም
☐ 5. በጣም እርካታ የለኝም

48. በሥራ ሰዓትዎ ምን ያህል እርካታ አለዎት?

☐ 1. በጣም እርካታ አለኝ
☐ 2. እርካታ አለኝ
☐ 3. መካከለኛ/ገለልተኛ
☐ 4. እርካታ የለኝም
☐ 5. በጣም እርካታ የለኝም

49. ከኃላፊዎ ጋር ባለዎት ግንኙነት ምን ያህል እርካታ አለዎት?

☐ 1. በጣም እርካታ አለኝ

☐ 2. እርካታ አለኝ

☐ 3. መካከለኛ/ገለልተኛ

☐ 4. እርካታ የለኝም

☐ 5. በጣም እርካታ የለኝም

50. ከሌሎች ሠራተኞች ጋር ባለዎት ግንኙነት ምን ያህል እርካታ አለዎት?

☐ 1. በጣም እርካታ አለኝ

☐ 2. እርካታ አለኝ

☐ 3. መካከለኛ/ገለልተኛ

☐ 4. እርካታ የለኝም

☐ 5. በጣም እርካታ የለኝም

ክፍል XI. የአካባቢ እርካታ

51. በአጠቃላይ በሥራ አካባቢዎ እርካታ አለዎት?

☐ 1. አዎ

☐ 2. አይ

52. በሥራ ቦታዎ ንጽህናና ደህንነት እርካታ አለዎት?

☐ 1. አዎ

☐ 2. አይ

53. በቂ የጽዳት ቁሳቁስና መሣሪያዎች መኖራቸው እርካታ ይሰጥዎታል?

☐ 1. አዎ

☐ 2. አይ

54. በሥራ ቦታዎ ያለው የአየር ማናፈሻ ሁኔታ እርካታ ይሰጥዎታል?

☐ 1. አዎ

☐ 2. አይ

55. በሥራ ቦታዎ ያለው የመብራት ሁኔታ እርካታ ይሰጥዎታል?

☐ 1. አዎ

☐ 2. አይ

56. የውሃና የንጽህና መጠበቂያ አገልግሎቶች መኖራቸው እርካታ ይሰጥዎታል?

☐ 1. አዎ

☐ 2. አይ

ክፍል XII. እውቅናና ማኅበራዊ ድጋፍ

57. እንደ ሆስፒታል ጽዳት ሠራተኛ የሚያከናውኑት ሥራ በሆስፒታሉ አስተዳደር በቂ እውቅና ይሰጠዋል ብለው ይሰማዎታል?

☐ 1. አዎ

☐ 2. አይ

58. የሆስፒታሉ ሠራተኞች ለሚያከናውኑት ሥራ ክብር ይሰጣሉ ብለው ይሰማዎታል?

☐ 1. አዎ

☐ 2. አይ

59. ሥራዎን በጥሩ ሁኔታ ሲያከናውኑ ምስጋና/አድናቆት ያገኛሉ?

☐ 1. አዎ

☐ 2. አይ

60. ለታካሚዎች ደህንነትና ለሆስፒታል ኢንፌክሽን መከላከል የሚያደርጉት አስተዋጽኦ እውቅና ይሰጠዋል ብለው ይሰማዎታል?

☐ 1. አዎ

☐ 2. አይ

61. ለሚያከናውኑት ሥራ በቂ ማኅበራዊ እውቅና አለመሰጠት ያጋጥምዎታል?

☐ 1. አዎ

☐ 2. አይ

ክፍል XIII. ተጨማሪ የሥራ ቦታ ምክንያቶች

62. የተመደቡልዎትን ሥራዎች ለማከናወን በቂ የጽዳት መሣሪያ አለዎት?

☐ 1. አዎ

☐ 2. አይ

63. በቂ የጽዳት አቅርቦት/ቁሳቁስ አለዎት?

☐ 1. አዎ

☐ 2. አይ

64. አካላዊ ጥረት የሚጠይቁ የጽዳት ሥራዎችን በተደጋጋሚ ያከናውናሉ?

☐ 1. አዎ

☐ 2. አይ

65. በሥራዎ ወቅት ደስ የማይል ሽታ ያጋጥምዎታል?

☐ 1. አዎ

☐ 2. አይ

66. ለኬሚካል የጽዳት ንጥረ ነገሮች ይጋለጣሉ?

☐ 1. አዎ

☐ 2. አይ

67. የጽዳት ሥራዎን በሚያከናውኑበት ጊዜ ደህንነት ይሰማዎታል?

☐ 1. አዎ

☐ 2. አይ

68. ከሥራ ጋር የተያያዘ ችግር ሲያጋጥምዎት ከቅርብ ኃላፊዎ በቂ ድጋፍ ያገኛሉ?

☐ 1. ሁልጊዜ

☐ 2. አንዳንድ ጊዜ

☐ 3. በፍጹም

ስለተሳተፉ ከልብ እናመሰግናለን።

7.1. Waraqaa Odeeffannoo fi Unka Hayyama Fedhii Guutuu Tajaajila Ol’aanaa Hospitaalota Bahaa Itoophiyaa keessatti gaggeeffamuuf (Vershinii Afaan Oromoo)

Seensa

Maqaan koo Sina Temesgen Tolera jedhama. Ani qorannoo mata-duree “Dhiphina Hojii Humnoota Hojii Irra Tuffatamanii Hospitaalota Tajaajila Ol’aanaa Bahaa Itoophiyaa Keessatti: Qorannoo Kutaalee Yeroo Tokkootti Gaggeeffamu” jedhu, Kolleejjii Saayinsii Fayyaa fi Meedikaalaa Yunivarsiitii Haramayaa keessatti gaggeeffamaa jiruuf Qorataa Ijoo (Principal Investigator) dha. Kaayyoo qorannichaa, adeemsa isaa fi hirmaannaa hojjettootaa qorannoo kana keessatti ilaalchisee akka isin hubattan isin gaafadha.

Mata-duree Qorannichaa

Dhiphina Hojii Humnoota Hojii Irra Tuffatamanii Hospitaalota Tajaajila Ol’aanaa Bahaa Itoophiyaa Keessatti: Qorannoo Kutaalee Yeroo Tokkootti Gaggeeffamu

Kaayyoo Qorannichaa

Kaayyoon ijoo qorannoo kanaa, hamma dhiphina hojii madaaluu fi wantoota dhiphina hojii waliin walqabatan hojjettoota hojii irra tuffatamanii hospitaalota tajaajila ol’aanaa Bahaa Itoophiyaa keessatti hojjetan gidduutti adda baasuu dha. Qorannoon kunis hojiiwwan barnootaa fi qorannoo Yunivarsiitii Haramayaa keessatti gaggeeffaman keessaa tokko dha.

Bu’aan qorannichaa bulchitoota hospitaalaa, hoggantoota damee fayyaa fi qaamolee dhimmi ilaallatuuf rakkoolee fi sababoota dhiphina bakka hojii hubachuu, akkasumas haalota hojii, fayyaa fi nageenya hojjettootaa fi oomishtummaa isaanii fooyyessuuf tarsiimoo sirrii qopheessuuf ragaa barbaachisaa ni kenna.

Adeemsa fi Yeroo Qorannichaa

Namoonni odeeffannoo walitti qaban gaaffilee qindaa’an fayyadamuun hojjettoota ulaagaalee qorannichaa guutan hospitaalota tajaajila ol’aanaa filataman keessatti hojjetan irraa odeeffannoo ni funaanu. Gaaffileen kun amala hawaas-dinagdee, haalota hojii, wantoota hojii waliin walqabatan, muuxannoo bakka hojii fi dhiphina hojii ilaalchisee gaaffilee ni qabaatu.

Gaaffiiwwan kana guutuun tilmaamaan daqiiqaa 30–40 fudhata. Kanaaf, dhaabbanni keessan qorannoon kun hojjettoota ulaagaalee guutan irratti akka gaggeeffamuuf eeyyama akka kennu kabajaan gaafanna.

Balaa fi Faayidaa

Qorannoo kana keessatti hirmaachuun balaa qaamaa guddaa qaba jedhamee hin yaadamne. Haa ta’u malee, gaaffileen tokko tokko muuxannoo bakka hojii ykn dhiphina hojii ilaalchisee dhiyaatan miira namaa xiqqoo jeequu ykn nama dhiphisuu danda’u. Hirmaataan gaaffii deebisuu hin barbaanne kamiyyuu deebisuu dhiisuu danda’a.

Qorannoo kana keessatti hirmaachuuf kaffaltiin maallaqaa ykn badhaasni kallattiin kennamu hin jiru.

Hirmaattonni faayidaa kallattii argachuu baatanillee, bu’aan qorannichaa hospitaalota tajaajila ol’aanaa, hoggantoota fayyaa, qaamolee imaammataa fi dhimmamtoota biroof rakkoolee bakka hojii adda baasuu, dhiphina hojii hir’isuu fi haala hojii fooyyessuuf tarkaanfiiwwan barbaachisan qopheessuuf odeeffannoo barbaachisaa ni kenna.

Iccitii Odeeffannoo

Odeeffannoon qorannoo kana keessatti walitti qabamu hundi sirriitti iccitii eegamee ni qabama. Maqaan hirmaataa ykn odeeffannoon eenyummaa isaa adda baasu kamiyyuu gabaasa qorannoo keessatti hin galmaa’u. Hirmaattonni maqaa isaanii yerineessanii koodii addaatiin ni beekamu.

Odeeffannoon argamu kaayyoo qorannoo qofaaf ni oola. Bu’aan qorannichaa bifa waliigalaatiin gabaafama; kanaaf hirmaataan dhuunfaa tokko eenyu akka ta’e adda baasuun hin danda’amu.

Mirga

Qorannoon kun dhaabbata keessan keessatti akka gaggeeffamuuf eeyyama kennuun guutumaan guutuutti fedhii irratti kan hundaa’e dha. Dhaabbata keessan bakka bu’uun qorannicha eeyyamuu ykn diduu mirga qabdu.

Yoo qorannicha eeyyamte, yeroo odeeffannoon walitti qabamu adeemsa dogoggoraa, sirrii hin taane ykn naamusa qorannoo cabsu yoo argite, qorannichi dhaabbata keessan keessatti akka dhaabbatu gaafachuu mirga qabda.

Qorannicha eeyyamuu diduun adabbii ykn faayidaa dhaabbataa dhabuu hin fidu.

Garee Qorannoo

- Sina Temesgen Tolera – Qorataa Ijoo
- Roba Argaw Tesema – Miseensa Garee Qorannoo
- Dawit Kassaye Getaneh – Miseensa Garee Qorannoo
- Ararso Tafesse – Miseensa Garee Qorannoo

Teessoo Qorataa Ijoo

Gaaffii ykn odeeffannoo dabalataa qorannicha ykn adeemsa isaa ilaalchisee yoo qabaattan, qunnamaa:

Sina Temesgen Tolera
Qorataa Ijoo
Bilbila: +251 913 023 634
Imeelii: sinetem3@gmail.com

Teessoo IHRERC

Institutional Health Research Ethics Review Committee (IHRERC)
Haramaya University, College of Health and Medical Sciences
Bilbila Waajjiraa: +251-254-66-2012
P.O. Box 235, Harar, Ethiopia

Ibsa Hayyama Fedhii Guutuu

Odeeffannoo waraqaa odeeffannoo kana keessatti kenname dubbisee hubadheera. Kaayyoo qorannichaa, adeemsa isaa, balaa fi faayidaa danda'amu, iccitii odeeffannoo, hirmaannaa fedhii irratti hundaa'e fi teessoo quunnamtii odeeffannoo dabalataa argachuuf barbaachisu sirriitti naaf ibsameera.

Gaaffiiwwan koo gaafachuuf carraan naaf kennameera, deebii naaf quubsaa ta'es argadheera.

Hirmaannaan qorannoo kanaa fedhii koo irratti kan hundaa'e ta'uu fi yeroo adeemsa odeeffannoo walitti qabuu keessatti wanta dogoggoraa, sirrii hin taane ykn naamusa qorannoo cabsu yoo arge, eeyyama koo yeroo kamiyyuu diduu ykn qorannicha dhaabsiisuu akka danda'u hubadheera.

Kanaaf, maqaa ______________________________ Hospitaala Tajaajila Ol'aanaa bakka bu'uun, qorannoon kun hojjettoota ulaagaalee guutan dhaabbaticha keessatti akka gaggeeffamuuf fedhiidhaan eeyyama nan kenna.

Maqaa fi mallattoo bakka bu'aa/bulchaa Hospitaalaa

Maqaa: ______________________________
Mallattoo: ___________________________
Guyyaa: ______________________________

Maqaa fi mallattoo Qorataa Ijoo

Maqaa: Sina Temesgen Tolera
Mallattoo: ___________________________
Guyyaa: ______________________________

7.2. Unka Hayyama Fedhii Guutuu Hirmaataa Qorannoo (> Umurii Waggaa 18)Vershinii Afaan Oromoo

Seensa

Maqaan koo ______________________________ jedhama. Ani qorannoo mata-duree "Dhiphina Hojii Humnoota Hojii Irra Tuffatamanii Hospitaalota Tajaajila Ol'aanaa Bahaa Itoophiyaa Keessatti: Qorannoo Kutaalee Yeroo Tokkootti Gaggeeffamu" jedhu, Sina Temesgen Tolera fi garee qorannoo Kolleejjii Saayinsii Fayyaa fi Meedikaalaa Yunivarsiitii Haramayaa keessatti gaggeessaa jiran keessatti nama odeeffannoo walitti qabuun hojjedha.

Kaayyoo qorannichaa fi hirmaannaa keessan keessatti maal akka isin irraa eegamu isiniif ibsuuf xiyyeeffannoo keessan akka naaf kennitan kabajaan isin gaafadha.

Mata-duree Qorannichaa

Dhiphina Hojii Humnoota Hojii Irra Tuffatamanii Hospitaalota Tajaajila Ol'aanaa Bahaa Itoophiyaa Keessatti: Qorannoo Kutaalee Yeroo Tokkootti Gaggeeffamu

Kaayyoo Qorannichaa

Kaayyoon ijoo qorannoo kanaa, hamma dhiphina hojii madaaluu fi wantoota dhiphina hojii waliin walqabatan hojjettoota hojii irra tuffatamanii hospitaalota tajaajila ol'aanaa Bahaa Itoophiyaa keessatti hojjetan gidduutti adda baasuu dha.

Bu'aan qorannichaa bulchitoota hospitaalaa, hoggantoota fayyaa fi qaamolee dhimmicha ilaallatan rakkoolee bakka hojii adda baasuu, haalota hojii fi nageenya hojjettootaa fooyyessuu, akkasumas qulqullina tajaajila kennamu fooyyessuuf tarsiimoo sirrii qopheessuuf ragaa ni kenna.

Adeemsa fi Yeroo Qorannichaa

Yoo qorannicha keessatti hirmaachuuf walii galtan, gaaffilee qindaa'an isiniif dhiyeessa. Gaaffileen kun amala hawaas-dinagdee, haalota hojii, wantoota hojii waliin walqabatan, muuxannoo bakka hojii fi dhiphina hojii ilaalchisee ta'u.

Gaaffilee kana guutuun tilmaamaan daqiiqaa 30–40 fudhata. Kanaaf, yeroo kana qorannicha keessatti hirmaachuuf akka nuuf kennitan kabajaan isin gaafanna.

Balaa fi Faayidaa

Qorannoo kana keessatti hirmaachuun balaa qaamaa guddaa akka fidu hin yaadamne. Haa ta'u malee, gaaffileen tokko tokko muuxannoo bakka hojii ykn dhiphina hojii ilaalchisee dhiyaatan miira namaa xiqqoo jeequu danda'u.

Gaaffii deebisuu hin barbaanne kamiyyuu deebisuu dhiisuu mirga qabdu.

Qorannicha keessatti hirmaachuuf kaffaltiin maallaqaa ykn badhaasni kallattiin hin jiru. Haa ta'u malee, bu'aan qorannichaa hospitaala keessanii fi qaamolee dhimmamtoota biroof dhiphina bakka hojii hubachuu fi haalota hojii fi fayyaa hojjettootaa fooyyessuuf tarkaanfiiwwan barbaachisan qopheessuuf odeeffannoo barbaachisaa ni kenna.

Iccitii Odeeffannoo

Odeeffannoon isin irraa walitti qabamu hundi sirriitti iccitii eegamee ni qabama. Maqaan keessan gabaasa qorannoo keessatti hin galmaa'u, odeeffannoon eenyummaa keessan kallattiin adda baasu kamiyyuu nama biraaf hin ibsamu.

Maqaa keessan bakka bu'uuf koodii adda ta'e fayyadamuun ni danda'ama. Odeeffannoon isin kennitan kaayyoo qorannoo qofaaf ni oola, bu'aan isaas bifa waliigalaatiin gabaafama.

Mirga

Qorannoo kana keessatti hirmaachuun guutumaan guutuutti fedhii keessan irratti hundaa'a. Hirmaachuu ykn hirmaachuu dhiisuu murteessuuf mirga guutuu qabdu.

Yoo hirmaachuuf walii galtan, sababaa tokko illee osoo hin kennin yeroo kamiyyuu qorannicha keessaa bahuu dandeessu. Akkasumas, gaaffii deebisuu hin barbaanne kamiyyuu deebisuu dhiisuu dandeessu.

Murteen keessan hirmaachuu, diduu ykn qorannicha keessaa bahuu ilaalchisee hojii keessan, mirga keessan, faayidaa keessan ykn hariiroo dhaabbata keessanii waliin qabdan irratti dhiibbaa hin fidu.

Garee Qorannoo

- Sina Temesgen Tolera – Qorataa Ijoo
- Roba Argaw Tesema – Miseensa Garee Qorannoo
- Dawit Kassaye Getaneh – Miseensa Garee Qorannoo
- Ararso Tafesse – Miseensa Garee Qorannoo

Teessoo Qorataa Ijoo

Sina Temesgen Tolera
Qorataa Ijoo
Bilbila: +251 913 023 634
Imeelii: sinetem3@gmail.com

Teessoo IHRERC

Institutional Health Research Ethics Review Committee (IHRERC)
Haramaya University, College of Health and Medical Sciences
Bilbila Waajjiraa: +251-254-66-2012
P.O. Box 235, Harar, Ethiopia

Ibsa Hayyama Fedhii Guutuu

Odeeffannoo waraqaa odeeffannoo kana keessatti kenname dubbisee hubadheera. Kaayyoo qorannichaa, adeemsa isaa, balaa fi faayidaa danda'amu, iccitii odeeffannoo, hirmaannaa fedhii irratti hundaa'e fi mirga koo akka hirmaataa qorannoo sirriitti naaf ibsameera.

Gaaffiiwwan koo gaafachuuf carraan naaf kennameera, gaaffiiwwan naaf hin galleef deebii quubsaa argadheera.

Hirmaannaan koo guutumaan guutuutti fedhii koo irratti kan hundaa'e ta'uu hubadheera. Gaaffii kamiyyuu deebisuu diduu ykn yeroo kamiyyuu sababaa tokko malee qorannicha keessaa bahuu nan danda'a; kana gochuunis mirga ykn faayidaa ani qabu na jalaa hin balleessu.

Kanaaf, mallattoo koo armaan gadiitiin, qorannoo mata-duree "Dhiphina Hojii Humnoota Hojii Irra Tuffatamanii Hospitaalota Tajaajila Ol'aanaa Bahaa Itoophiyaa Keessatti: Qorannoo Kutaalee Yeroo Tokkootti Gaggeeffamu" jedhu keessatti fedhiidhaan hirmaachuuf walii nan gala.

Maqaa fi mallattoo Hirmaataa Qorannoo

Maqaa: ________________________________
Mallattoo: _____________________________
Guyyaa: ________________________________

Maqaa fi mallattoo Nama Odeeffannoo Walitti Qabu

Maqaa: ________________________________
Mallattoo: _____________________________
Guyyaa: ________________________________

7.3. Gaaffilee Qorannoo: Dhiphina Hojii Qorattoota/Qulqulleessitoota Hospitaalaa Bahaa Itoophiyaa Keessatti

Seensa Hirmaataaf

Kabajamaa hirmaataa,

Gaaffileen kun dhiphina hojii, haalota hojii, wantoota amala namaa fi wantoota naannoo waliin walqabatan qulqulleessitoota hospitaalaa gidduutti madaaluuf qophaa’aniiru.

Deebiin keessan hundi iccitii eegamee kan qabamu yoo ta’u, kaayyoo qorannoo qofaaf ni oola. Deebiin sirrii ykn dogoggoraa jedhu hin jiru. Mee gaaffiiwwan hunda muuxannoo keessan irratti hundaa’uun dhugaa fi iftoomina qabuun deebisaa.

Qajeelfama: Gaaffilee baay’eef, deebii haala keessan sirriitti ibsu bira mallattoo (✓) kaa’aa.

Kutaa I. Amaloota Hawaas-Dimogiraafii fi Hojii

1. Saala

Saalli keessan maali?

☐ 1. Dubartii
☐ 2. Dhiira

2. Umurii

Umuriin keessan meeqa? ______ waggaa

Qorannoo keessatti:

☐ 1. Waggaa 24 gadi
☐ 2. Waggaa 25–35
☐ 3. Waggaa 35 ol

3. Haala Gaa’elaa

☐ 1. Qoma
☐ 2. Fuudheera/Heerumeera
☐ 3. Addaan ba’eera/Hiikeera

4. Sadarkaa Barnootaa Olaanaa Xumurame

☐ 1. Kutaa 5 gadi
☐ 2. Kutaa 5–8
☐ 3. Kutaa 8 ol

5. Muuxannoo Hojii Qulqulleessaa Hospitaalaa Waliigalaa

Qulqulleessaa taatanii waggaa meeqa hojjetaniittu?

_____ waggaa

Qorannoo keessatti:

☐ 1. Waggaa 2 gadi
☐ 2. Waggaa 3–5
☐ 3. Waggaa 5 ol

6. Gosa Qacarrii

☐ 1. Dhaabbataa
☐ 2. Kontiraataa

7. Mindaa Ji’aa

Mindaan ji’aa keessan tilmaamaan doolaara Ameerikaa meeqa?

$ ________

Qorannoo keessatti:

☐ 1. $21 gadi
☐ 2. $21–44
☐ 3. $44 ol

Yoo daataan Birrii Itoophiyaatiin walitti qabame, mindaa dhugaa ETB keessatti galmeessaa; sana booda safartuu jijjiirraa maallaqaa pirootokoolii qorannicha keessatti ibsame fayyadamuun gara USDtti jijjiiraa.

8. Lakkoofsa Shiftii Hojii Guyyaatti

☐ 1. Shiftii tokko
☐ 2. Shiftii lama
☐ 3. Shiftii sadii

Kutaa II. To’annoo Bakka Hojii fi Fayyaa fi Nageenya Hojii

9. Hojii qulqulleessuu keessan ilaalchisee hogganaan keessan isin yeroo meeqa to’ata?

☐ 1. To’annoon hin jiru
☐ 2. Yeroo tokko tokko
☐ 3. Yeroo hunda/Idilee

10. Hojii qulqulleessuu keessan waliin walqabatee leenjii Fayyaa fi Nageenya Hojii (OHS) argattanii beektuu?

☐ 1. Eeyyee
☐ 2. Lakki

11. Yoo eeyyee ta’e, yeroo dhiyoo keessatti leenjii OHS yoom argattan?

☐ 1. Ji’oota 6 darban keessatti
☐ 2. Ji’a 7–12 dura
☐ 3. Waggaa 1 ol dura
☐ 4. Hin yaadadhu

12. Bakki hojii keessan meeshaalee ittisaa dhuunfaa (PPE) gahaa isiniif ni kennaa?

☐ 1. Eeyyee
☐ 2. Lakki

13. Yeroo PPE isin barbaachisutti meeshaa isin barbaachisu argachuu ni dandeessuu?

☐ 1. Yeroo hunda
☐ 2. Yeroo tokko tokko
☐ 3. Gonkumaa

Kutaa III. Baay’ina Hojii fi Sa’aatii Hojii

14. Baay’inni hojii isin yeroo ammaa qabdan ulfaataa dha jettanii yaadduu?

☐ 1. Eeyyee
☐ 2. Lakki

15. Giddu-galeessaan guyyaatti sa’aatii meeqa hojjetu?

_____ sa’aatii/guyyaa

16. Guyyaatti yeroo baay’ee sa’aatii 8 ol hojjetuu?

☐ 1. Eeyyee
☐ 2. Lakki

17. Sa’aatii hojii keessan qophaa’e olitti hojjechuun isin irraa yeroo meeqa eegama?

☐ 1. Gonkumaa
☐ 2. Darbee darbee
☐ 3. Yeroo tokko tokko
☐ 4. Yeroo baay’ee
☐ 5. Yeroo hunda

18. Hojii qulqulleessuu isinitti ramadame xumuruuf yeroo gahaa qabduu?

☐ 1. Eeyyee
☐ 2. Lakki

19. Baay’inni hojii qulqulleessuu isinitti ramadame baay’ee ol’aanaa dha jettanii yaadduu?

☐ 1. Eeyyee
☐ 2. Lakki

Kutaa IV. Dhiphina Hojii

20. Ji'a darbe keessatti, hojii qulqulleessaa hospitaalaa taatanii hojjechuu keessaniin dhiphina hojii mudatanii beektuu?

☐ 1. Eeyyee
☐ 2. Lakki

Koodii:

Eeyyee = 1
Lakki = 0

21. Yoo eeyyee ta'e, maddoonni ijoo dhiphina hojii keessanii maal fa'i?

Deebii hedduu filachuun ni danda'ama.

☐ 1. Baay'ina hojii guddaa
☐ 2. Sa'aatii hojii dheeraa
☐ 3. Hojjettoota gahaa dhabuu
☐ 4. To'annoo hojii ga'aa dhabuu
☐ 5. Hojii koo sirriitti beekamtii dhabuu
☐ 6. Mindaa xiqqaa
☐ 7. Haala hojii gaarii hin taane
☐ 8. PPE dhabuu
☐ 9. Wantoota balaa geessisanitti saaxilamuu
☐ 10. Walitti bu'iinsa hojjettoota waliin
☐ 11. Walitti bu'iinsa hoggantoota waliin
☐ 12. Kan biraa: ____________

Kutaa V. Mallattoolee Qaamaa fi Sammuu Dhiphina Hojii Waliin Walqabatan

Qajeelfama: Gaaffii 20 irratti Eeyyee yoo jettan, ji'a darbe keessatti mallattoowwan armaan gadii yeroo meeqa ykn hamma kamiin akka isin mudatan agarsiisaa.

Lak.	Mallattoo	Hin jiru	Salphaa	Giddu-galeessa	Cimaa
22	Qaama hollachuu/raafamuu	☐	☐	☐	☐
23	Mataa naanna'uu	☐	☐	☐	☐

Lak.	Mallattoo	Hin jiru	Salphaa	Giddu-galeessa	Cimaa
24	Hanga malee dafquu	☐	☐	☐	☐
25	Dhukkubbii maashaa	☐	☐	☐	☐
26	Mataa dhukkubbii	☐	☐	☐	☐
27	Dugda dhukkubbii	☐	☐	☐	☐
28	Hirriba jeeqamuu	☐	☐	☐	☐
29	Hafuura baafachuu dadhabuu/rakkoo hafuuraa	☐	☐	☐	☐
30	Nyaata bulchuu dadhabuu/rakkoo garaacha	☐	☐	☐	☐
31	Dadhabbii ykn humna dhumuu	☐	☐	☐	☐
32	Ariifachuu/aaruu salphaa	☐	☐	☐	☐
33	Xiyyeeffannoo kennuu dadhabuu	☐	☐	☐	☐

Koodii yaadame:

0 = Hin jiru
1 = Salphaa
2 = Giddu-galeessa
3 = Cimaa

Kutaa VI. Hirriba

34. Ji’a darbe keessatti rakkoo ykn jeequmsa hirribaa mudatanii beektuu?

☐ 1. Eeyyee
☐ 2. Lakki

35. Yoo eeyyee ta’e, rakkoon hirribaa isin mudate maal ture?

☐ 1. Rafuu jalqabuu dadhabuu
☐ 2. Halkan keessa yeroo baay’ee dammaquu
☐ 3. Yeroo malee barii dammaquu
☐ 4. Hirriba qulqullina gaarii hin qabne
☐ 5. Kan biraa: ____________

36. Giddu-galeessaan halkan tokko keessatti sa’aatii meeqa rafuu?

_____ sa’aatii

Kutaa VII. Fayyadama Alkoolii

37. Yeroo ammaa dhugaatii alkoolii fayyadamtuu?

☐ 1. Eeyyee
☐ 2. Lakki

38. Guyyoota 30 darban keessatti alkoolii yeroo meeqa fayyadamtan?

☐ 1. Gonkumaa
☐ 2. Torbanitti yeroo tokko gadi
☐ 3. Torbanitti guyyaa 1–2
☐ 4. Torbanitti guyyaa 3–4
☐ 5. Guyyaa 5 ykn isaa ol torbanitti

39. Yeroo alkoolii dhugdan, fayyadama keessan baay’ee/ulfaataa dha jettanii ibsituu?

☐ 1. Eeyyee
☐ 2. Lakki

Qorannoo ijoo keessatti:

Fayyadama alkoolii ulfaataa: Eeyyee/Lakki

Kutaa VIII. Fayyadama Jimaa

40. Yeroo ammaa jimaa (chat) qamaa?

☐ 1. Eeyyee
☐ 2. Lakki

41. Guyyoota 30 darban keessatti jimaa yeroo meeqa qamtan?

☐ 1. Gonkumaa
☐ 2. Torbanitti yeroo tokko gadi
☐ 3. Torbanitti guyyaa 1–2
☐ 4. Torbanitti guyyaa 3–4
☐ 5. Guyyaa 5 ykn isaa ol torbanitti

42. Guyyaa jimaa qamtan tokko keessatti yeroo baay’ee sa’aatii meeqa jimaa qamaa?

_____ sa’aatii

Kutaa IX. Tamboo/Sigaaraa Xuuxuu

43. Yeroo ammaa sigaaraa xuuxxuu?

☐ 1. Eeyyee
☐ 2. Lakki

44. Darbe keessatti yeroo idileetti sigaaraa xuuxaa turtanii beektuu?

☐ 1. Eeyyee
☐ 2. Lakki

45. Yoo yeroo ammaa sigaaraa xuuxaa jirtan, guyyaatti tilmaamaan sigaaraa meeqa xuuxxu?

_____ sigaaraa

Kutaa X. Quufa Hojii

46. Walumaagalatti, hojii qulqulleessaa hospitaalaa yeroo ammaa qabdanitti quuftanii jirtuu?

☐ 1. Eeyyee
☐ 2. Lakki

47. Mindaa keessaniin hangam quuftuu?

☐ 1. Baay'ee quufa
☐ 2. Nan quufa
☐ 3. Giddu-galeessa
☐ 4. Hin quufu
☐ 5. Baay'ee hin quufu

48. Sa'aatii hojii keessaniin hangam quuftuu?

☐ 1. Baay'ee quufa
☐ 2. Nan quufa
☐ 3. Giddu-galeessa
☐ 4. Hin quufu
☐ 5. Baay'ee hin quufu

49. Hariiroo keessan hogganaa waliin qabdanitti hangam quuftuu?

☐ 1. Baay'ee quufa
☐ 2. Nan quufa
☐ 3. Giddu-galeessa
☐ 4. Hin quufu
☐ 5. Baay'ee hin quufu

50. Hariiroo keessan hojjettoota biroo waliin qabdanitti hangam quuftuu?

☐ 1. Baay’ee quufa
☐ 2. Nan quufa
☐ 3. Giddu-galeessa
☐ 4. Hin quufu
☐ 5. Baay’ee hin quufu

Kutaa XI. Quufa Naannoo Hojii

51. Walumaagalatti, naannoo hojii keessaniin quuftanii jirtuu?

☐ 1. Eeyyee
☐ 2. Lakki

52. Qulqullinaa fi nageenya bakka hojii keessaniin quuftanii jirtuu?

☐ 1. Eeyyee
☐ 2. Lakki

53. Argamummaa meeshaalee fi mi’a qulqulleessuu irratti quuftanii jirtuu?

☐ 1. Eeyyee
☐ 2. Lakki

54. Qilleensa seensisuun/ventileeshinii bakka hojii keessanii isin quubaa?

☐ 1. Eeyyee
☐ 2. Lakki

55. Ifa bakka hojii keessanii irratti quuftanii jirtuu?

☐ 1. Eeyyee
☐ 2. Lakki

56. Argamummaa bishaanii fi tajaajila qulqullinaa irratti quuftanii jirtuu?

☐ 1. Eeyyee
☐ 2. Lakki

Kutaa XII. Beekamtii fi Deeggarsa Hawaasummaa

57. Hojii keessan akka qulqulleessaa hospitaalaa bulchiinsi hospitaalaa sirriitti beekamtii kenneera jettanii yaadduu?

☐ 1. Eeyyee
☐ 2. Lakki

58. Hojjettoonni hospitaalaa hojii isin hojjetan kabaju jettanii yaadduu?

☐ 1. Eeyyee
☐ 2. Lakki

59. Yeroo hojii keessan sirriitti hojjetan galateeffannaa ykn jajjabeessa argattuu?

☐ 1. Eeyyee
☐ 2. Lakki

60. Gumaachi keessan nageenya dhukkubsataa fi ittisa infekshinii hospitaalaa keessatti qabdan beekamtii argata jettanii yaadduu?

☐ 1. Eeyyee
☐ 2. Lakki

61. Hojii keessaniif beekamtii hawaasummaa gahaa dhabuu mudatara?

☐ 1. Eeyyee
☐ 2. Lakki

Kutaa XIII. Wantoota Dabalataa Bakka Hojii

62. Hojii keessan raawwachuuf meeshaalee qulqulleessuu gahaa qabduu?

☐ 1. Eeyyee
☐ 2. Lakki

63. Meeshaalee/mi'a qulqulleessuu gahaa qabduu?

☐ 1. Eeyyee
☐ 2. Lakki

64. Yeroo baay'ee hojii qulqulleessuu qaamaan ulfaataa ta'e raawwattuu?

☐ 1. Eeyyee
☐ 2. Lakki

65. Yeroo hojii keessanii foolii hin jaallatamneef saaxilamtuu?

☐ 1. Eeyyee
☐ 2. Lakki

66. Keemikaalota qulqulleessuu keessatti fayyadamanitti saaxilamtuu?

☐ 1. Eeyyee
☐ 2. Lakki

67. Hojii qulqulleessuu keessan yeroo raawwattan nageenya keessaniin of eeggamaa ta'uu isinitti dhaga'amaa?

☐ 1. Eeyyee
☐ 2. Lakki

68. Yeroo rakkoon hojii isin mudatu, hogganaa keessan irraa deeggarsa gahaa argattuu?

☐ 1. Yeroo hunda
☐ 2. Yeroo tokko tokko
☐ 3. Gonkumaa

Qorannoo kana keessatti **hirmaannaa keessaniif galatoomaa!**

7.1. Xaashida Macluumaadka iyo Foomka Oggolaanshaha Ikhtiyaariga ah ee Isbitaallada Heerka Saddexaad ee Bariga Itoobiya

Hordhac

Magacaygu waa Sina Temesgen Tolera. Waxaan ahay Baaraha Guud (Principal Investigator) ee daraasadda cinwaankeedu yahay "Walbahaarka La Xiriira Shaqada ee Shaqaalaha Dayacan ee Ka Shaqeeya Isbitaallada Heerka Saddexaad ee Bariga Itoobiya: Daraasad Isdhaafsi ah (Cross-Sectional Study)", taas oo ay waddo Jaamacadda Haramaya, Kulliyadda Caafimaadka iyo Culuumta Caafimaadka.

Waxaan si xushmad leh idiinka codsanayaa inaad waqti naga siiso si aan idiinku sharaxo ujeeddada daraasadda, habraacyada ku lug leh, iyo ka-qaybgalka shaqaalaha ee cilmi-baaristan.

Cinwaanka Daraasadda

Walbahaarka La Xiriira Shaqada ee Shaqaalaha Dayacan ee Ka Shaqeeya Isbitaallada Heerka Saddexaad ee Bariga Itoobiya: Daraasad Isdhaafsi ah

Ujeeddada Daraasadda

Ujeeddada ugu weyn ee daraasaddan waa in la qiimeeyo heerka walbahaarka la xiriira shaqada iyo in la aqoonsado arrimaha la xiriira walbahaarka shaqada ee ka dhex jira shaqaalaha dayacan ee ka shaqeeya isbitaallada heerka saddexaad ee Bariga Itoobiya.

Daraasaddan sidoo kale waxaa loo sameynayaa qayb ka mid ah hawlaha tacliimeed iyo cilmi-baariseed ee Jaamacadda Haramaya. Natiijooyinka daraasaddu waxay siin karaan caddayn waxtar leh maamulayaasha isbitaallada, maareeyayaasha waaxda caafimaadka iyo daneeyayaasha kale, si ay u fahmaan waxyaabaha keena walbahaarka goobta shaqada iyo inay horumariyaan xeelado ku habboon oo lagu hagaajinayo xaaladaha shaqada, fayo-qabka iyo wax-soo-saarka shaqaalaha.

Habraaca iyo Muddada

Xog-ururiyeyaashu waxay shaqaalaha u qalma ee ka shaqeeya isbitaallada heerka saddexaad ee la doortay siin doonaan su'aalo habaysan. Su'aaluhu waxay ku saabsan doonaan:

- Astaamaha bulshada iyo tirakoobka;
- Xaaladaha shaqada;
- Arrimaha shaqada;
- Khibradaha goobta shaqada; iyo
- Walbahaarka la xiriira shaqada.

Buuxinta su'aalaha waxay qaadan doontaa qiyaastii 30–40 daqiiqo.

Sidaas darteed, waxaan si xushmad leh uga codsanaynaa hay'addiinna inay oggolaato in daraasaddan lagu sameeyo shaqaalaha u qalma.

Khataraha iyo Faa'iidooyinka

Lama filayo khatar jireed oo weyn oo ka dhalan karta ka-qaybgalka daraasaddan. Si kastaba ha ahaatee, su'aalaha qaarkood ee ku saabsan khibradaha goobta shaqada ama walbahaarka waxay keeni karaan raaxo-darro nafsiyeed oo yar ama dhib yar.

Ka-qaybgalayaashu waxay xaq u leeyihiin inay diidaan inay ka jawaabaan su'aal kasta oo aysan doonayn inay ka jawaabaan.

Ka-qaybgalka daraasaddan laguma siin doono lacag ama abaalmarin toos ah.

Inkasta oo ka-qaybgalayaashu aysan heli karin faa'iido toos ah, natiijooyinka daraasaddu waxay siin karaan isbitaallada heerka saddexaad, maareeyayaasha caafimaadka, siyaasad-dejiyeyaasha iyo daneeyayaasha kale macluumaad muhiim ah oo lagu aqoonsado caqabadaha goobta shaqada, laguna horumariyo faragelinno lagu yareynayo walbahaarka la xiriira shaqada iyo lagu hagaajinayo deegaanka shaqada.

Asturnaanta iyo Sirta

Dhammaan macluumaadka la ururiyo inta lagu jiro daraasaddan si adag ayaa loo ilaalin doonaa. Magaca ka-qaybgale ama macluumaad kale oo si gaar ah qofka lagu aqoonsan karo laguma dari doono warbixinta cilmi-baarista.

Ka-qaybgalayaasha waxaa lagu aqoonsan doonaa koodh, halkii magacyadooda laga isticmaali lahaa. Macluumaadka la helo waxaa loo isticmaali doonaa oo keliya ujeeddooyin cilmi-baariseed, natiijooyinkana waxaa loo soo bandhigi doonaa qaab guud si aan qof gaar ah loo aqoonsan karin.

Xuquuqda

Oggolaanshaha in daraasaddan lagu sameeyo hay'addiinna waa ikhtiyaari buuxa. Waxaad xaq u leedahay inaad oggolaato ama diido daraasaddan adigoo matalaya hay'addiinna.

Haddii aad oggolaato daraasadda, waxaad xaq u leedahay inaad codsato in daraasadda lagu joojiyo hay'addiinna haddii aad aragto habraacyo marin-habaabin ah, aan habboonayn ama aan waafaqsanayn anshaxa cilmi-baarista inta lagu jiro xog-ururinta.

Diidmada oggolaanshaha daraasaddan ma keenayso ciqaab ama luminta wax faa'iido ah oo hay'addu xaq u leedahay.

Kooxda Cilmi-baarista

- Sina Temesgen Tolera – Baaraha Guud
- Roba Argaw Tesema – Xubin Kooxda Cilmi-baarista
- Dawit Kassaye Getaneh – Xubin Kooxda Cilmi-baarista
- Ararso Tafesse – Xubin Kooxda Cilmi-baarista

Cinwaanka Xiriirka Baaraha Guud

Haddii aad qabto wax su'aalo ah ama aad u baahan tahay macluumaad dheeraad ah oo ku saabsan daraasadda ama habraacyadeeda, fadlan la xiriir:

Sina Temesgen Tolera
Baaraha Guud
Mobile: +251 913 023 634
Email: sinetem3@gmail.com

Cinwaanka Xiriirka IHRERC

Institutional Health Research Ethics Review Committee (IHRERC)
Haramaya University, College of Health and Medical Sciences
Office Phone: +251-254-66-2012
P.O. Box 235, Harar, Ethiopia

Caddeynta Oggolaanshaha Ikhtiyaariga ah ee La Fahmay

Waxaan akhriyey oo fahmay macluumaadka lagu bixiyey xaashidan macluumaadka. Waxaa si cad la ii sharaxay ujeeddada daraasadda, habraacyada, khataraha iyo faa'iidooyinka suurtagalka ah, sirta macluumaadka, ka-qaybgalka ikhtiyaariga ah iyo cinwaannada xiriirka ee wixii su'aalo ah.

Waxaa la ii oggolaaday inaan su'aalo weydiiyo, waxaana helay jawaabo ku filan oo ku saabsan su'aalahayga.

Waxaan fahmay in ka-qaybgalka daraasaddu yahay mid ikhtiyaari ah, isla markaana aan xaq u leeyahay inaan diido ama ka noqdo oggolaanshahayga wakhti kasta haddii aan arko habraacyo marin-habaabin ah, aan habboonayn ama aan waafaqsanayn anshaxa cilmi-baarista inta lagu jiro xog-ururinta.

Sidaas darteed, waxaan si ikhtiyaari ah oggolaansho uga bixinayaa magaca ______________________________ Isbitaalka Heerka Saddexaad in daraasaddan lagu sameeyo shaqaalaha u qalma ee hay'adda.

Magaca iyo saxiixa wakiilka/madaxa isbitaalka:

Magac: ______________________________
Saxiix: ______________________________
Taariikh: ______________________________

Magaca iyo saxiixa Baaraha Guud:

Magac: Sina Temesgen Tolera
Saxiix: ______________________________
Taariikh: ______________________________

7.2. Foomka Oggolaanshaha Ikhtiyaariga ah ee Ka-qaybgalayaasha Daraasadda (>18 sano)

Hordhac

Magacaygu waa ______________________________. Waxaan ahay xog-ururiye ka shaqaynaya daraasadda cinwaankeedu yahay "Walbahaarka La Xiriira Shaqada ee Shaqaalaha Dayacan ee Ka Shaqeeya Isbitaallada Heerka Saddexaad ee Bariga Itoobiya: Daraasad Isdhaafsi ah", taas oo ay waddaan Sina Temesgen Tolera iyo kooxda cilmi-baarista ee Jaamacadda Haramaya, Kulliyadda Caafimaadka iyo Culuumta Caafimaadka.

Waxaan si xushmad leh kuugu codsanayaa inaad waqti yar na siiso si aan kuugu sharaxo daraasadda iyo ka-qaybgalkaaga.

Cinwaanka Daraasadda

Walbahaarka La Xiriira Shaqada ee Shaqaalaha Dayacan ee Ka Shaqeeya Isbitaallada Heerka Saddexaad ee Bariga Itoobiya: Daraasad Isdhaafsi ah

Ujeeddada Daraasadda

Ujeeddada ugu weyn ee daraasaddan waa in la qiimeeyo heerka walbahaarka la xiriira shaqada iyo in la aqoonsado arrimaha la xiriira walbahaarka shaqada ee shaqaalaha dayacan ee ka shaqeeya isbitaallada heerka saddexaad ee Bariga Itoobiya.

Natiijooyinka daraasaddu waxay siin doonaan caddayn ka caawin karta maamulayaasha isbitaallada, maareeyayaasha caafimaadka iyo daneeyayaasha kale inay aqoonsadaan caqabadaha goobta shaqada iyo inay horumariyaan xeelado ku habboon oo lagu hagaajinayo xaaladaha shaqada, fayo-qabka shaqaalaha iyo tayada adeegyada la bixiyo.

Habraaca iyo Muddada

Haddii aad oggolaato inaad ka-qaybqaadato, waxaan ku siin doonaa su'aalo habaysan. Su'aaluhu waxay ku saabsan yihiin:

- Astaamaha bulshada iyo tirakoobka;
- Xaaladaha shaqada;
- Arrimaha shaqada;
- Khibradaha goobta shaqada; iyo
- Walbahaarka la xiriira shaqada.

Buuxinta su'aalaha waxay qaadan doontaa qiyaastii 30–40 daqiiqo. Waxaan si xushmad leh kaaga codsanayaa inaad waqtigan u hurto ka-qaybgalka daraasadda.

Khataraha iyo Faa'iidooyinka

Lama filayo khatar jireed oo weyn oo ka dhalata ka-qaybgalka daraasaddan. Si kastaba ha ahaatee, su'aalaha qaarkood ee ku saabsan khibradaha goobta shaqada ama walbahaarka waxay keeni karaan raaxo-darro yar.

Waxaad xaq u leedahay inaad ka boodo su'aal kasta oo aadan doonayn inaad ka jawaabto.

Ka-qaybgalka daraasaddan laguma siin doono lacag ama abaalmarin toos ah. Si kastaba ha ahaatee, natiijooyinka daraasaddu waxay siin karaan isbitaalkaaga iyo daneeyayaasha kale macluumaad waxtar leh oo ku saabsan walbahaarka goobta shaqada iyo tallaabooyinka lagu hagaajin karo xaaladaha shaqada iyo fayo-qabka shaqaalaha.

Asturnaanta iyo Sirta

Dhammaan macluumaadka aad bixiso si adag ayaa loo ilaalin doonaa. Magacaaga laguma qori doono warbixinta cilmi-baarista, mana la shaacin doono wax macluumaad ah oo si toos ah kuu aqoonsan kara.

Koodh gaar ah ayaa loo isticmaali karaa halkii magacaaga laga isticmaali lahaa. Macluumaadka aad bixiso waxaa loo isticmaali doonaa oo keliya ujeeddooyin cilmi-baariseed, natiijooyinkana waxaa loo soo bandhigi doonaa qaab guud.

Xuquuqda

Ka-qaybgalka daraasaddan waa ikhtiyaari buuxa. Waxaad xaq u leedahay inaad go'aansato inaad ka-qaybqaadato iyo in kale.

Haddii aad oggolaato inaad ka-qaybqaadato, waxaad xaq u leedahay inaad daraasadda ka baxdo wakhti kasta adigoon sabab bixin. Sidoo kale waxaad diidi kartaa inaad ka jawaabto su'aal kasta oo aadan doonayn inaad ka jawaabto.

Go'aankaaga inaad ka-qaybqaadato, diido ama ka baxdo daraasadda wax saameyn ah kuma yeelan doono shaqadaada, xuquuqdaada, faa'iidooyinkaaga ama xiriirka aad la leedahay hay'addaada.

Kooxda Cilmi-baarista

- Sina Temesgen Tolera – Baaraha Guud
- Roba Argaw Tesema – Xubin Kooxda Cilmi-baarista
- Dawit Kassaye Getaneh – Xubin Kooxda Cilmi-baarista
- Ararso Tafesse – Xubin Kooxda Cilmi-baarista

Cinwaanka Xiriirka Baaraha Guud

Sina Temesgen Tolera
Baaraha Guud
Mobile: +251 913 023 634
Email: sinetem3@gmail.com

Cinwaanka Xiriirka IHRERC

Institutional Health Research Ethics Review Committee (IHRERC)
Haramaya University, College of Health and Medical Sciences
Office Phone: +251-254-66-2012
P.O. Box 235, Harar, Ethiopia

Caddeynta Oggolaanshaha La Fahmay

Waxaan akhriyey oo fahmay macluumaadka lagu bixiyey xaashidan macluumaadka. Waxaa la ii sharaxay ujeeddada daraasadda, habraacyada, khataraha iyo faa'iidooyinka suurtagalka ah, sirta macluumaadka, ka-qaybgalka ikhtiyaariga ah iyo xuquuqdayda ka-qaybgale ahaan.

Waxaa la ii oggolaaday inaan su'aalo weydiiyo, waxaana helay jawaabo ku filan oo ku saabsan su'aalihii aan qabay.

Waxaan fahmay in ka-qaybgalka daraasaddu yahay mid ikhtiyaari ah. Waxaan fahmay inaan diidi karo inaan ka jawaabo su'aal kasta ama aan daraasadda ka bixi karo wakhti kasta, anigoon lumin wax xuquuq ama faa'iido ah oo aan xaq u leeyahay.

Sidaas darteed, waxaan si ikhtiyaari ah u oggolaanayaa inaan ka-qaybqaato daraasadda cinwaankeedu yahay "Walbahaarka La Xiriira Shaqada ee Shaqaalaha Dayacan ee Ka Shaqeeya Isbitaallada Heerka Saddexaad ee Bariga Itoobiya: Daraasad Isdhaafsi ah," sida uu muujinayo saxiixayga hoose.

Magaca iyo saxiixa Ka-qaybgalaha Daraasadda:

Magac: ______________________________
Saxiix: ______________________________
Taariikh: ____________________________

Magaca iyo saxiixa Xog-ururiyaha:

Magac: ______________________________
Saxiix: ______________________________
Taariikh: ____________________________

7.3. Su'aalaha Daraasadda: Walbahaarka La Xiriira Shaqada ee Nadiifiyeyaasha Isbitaallada ee Bariga Itoobiya

Hordhac ku socda Ka-qaybgalaha

Ka-qaybgale qaaliga ah,

Su'aalahan waxaa loogu talagalay in lagu qiimeeyo walbahaarka la xiriira shaqada, xaaladaha shaqada, arrimaha hab-dhaqanka iyo arrimaha deegaanka ee nadiifiyeyaasha isbitaallada.

Jawaabaha aad bixiso si qarsoodi ah ayaa loo ilaalin doonaa, waxaana loo isticmaali doonaa oo keliya ujeeddooyin cilmi-baariseed.

Ma jiraan jawaabo sax ah ama khaldan. Fadlan su'aal kasta si daacad ah uga jawaab adigoo ku salaynaya khibraddaada.

Tilmaamaha: Su'aalaha badankooda, fadlan ku calaamadee (✓) jawaabta sida ugu fiican u qeexaysa xaaladdaada.

QAYBTA I. Astaamaha Bulshada, Tirakoobka iyo Shaqada

1. Jinsiga

Waa maxay jinsigaagu?

☐ 1. Dumar
☐ 2. Rag

2. Da'da

Immisa jir baad tahay? ______ sano

Falanqaynta:

☐ 1. Ka yar 24 sano
☐ 2. 25–35 sano
☐ 3. Ka weyn 35 sano

3. Xaaladda Guurka

☐ 1. Aan guursan
☐ 2. Guursaday/Guursatay
☐ 3. Kala maqnaa/Furay

4. Heerka Waxbarasho ee Ugu Sarreeya ee Aad Dhammaysay

☐ 1. Ka hooseeya fasalka 5aad
☐ 2. Fasalka 5aad–8aad
☐ 3. Ka sarreeya fasalka 8aad

5. Wadarta Khibradda Shaqo ee Aad U Leedahay Nadiifinta Isbitaalka

Immisa sano ayaad nadiifiye ahaan u shaqaynaysay?

_____ sano

Falanqaynta:

☐ 1. Ka yar 2 sano
☐ 2. 3–5 sano
☐ 3. Ka badan 5 sano

6. Nooca Shaqada

☐ 1. Shaqo joogto ah
☐ 2. Shaqo qandaraas ah

7. Mushaharka Bishii

Qiyaastii waa imisa mushaharkaaga bishii ee doollarka Maraykanka?

$ ________

Falanqaynta:

☐ 1. Ka yar $21
☐ 2. $21–44
☐ 3. Ka badan $44

Haddii xogta lagu ururiyo Birr Itoobiya, qor mushaharka dhabta ah ee ETB, kadibna USD ugu beddel adigoo adeegsanaya heerka sarrifka lacagta ee lagu qeexay habraaca daraasadda.

8. Tirada Shaftsiga Shaqada Maalintii

☐ 1. Hal shift
☐ 2. Laba shift
☐ 3. Saddex shift

QAYBTA II. Kormeerka Goobta Shaqada iyo Caafimaadka iyo Badbaadada Shaqada

9. Immisa jeer ayuu kormeerahaaga tooska ahi kuu kormeeraa shaqada nadiifinta?

☐ 1. Kormeer ma jiro
☐ 2. Mararka qaarkood
☐ 3. Si joogto ah

10. Weligaa ma heshay tababar ku saabsan Caafimaadka iyo Badbaadada Shaqada (OHS) oo la xiriira shaqadaada nadiifinta?

☐ 1. Haa
☐ 2. Maya

11. Haddii Haa, goormaad ugu dambaysay tababarka OHS?

☐ 1. 6dii bilood ee la soo dhaafay
☐ 2. 7–12 bilood ka hor
☐ 3. In ka badan hal sano ka hor
☐ 4. Ma xasuusto

12. Goobta shaqadu ma ku siisaa qalabka ilaalinta shaqsiga (PPE) oo ku filan?

☐ 1. Haa
☐ 2. Maya

13. Ma heli kartaa PPE-ga aad u baahan tahay mar kasta oo aad u baahato?

☐ 1. Had iyo jeer
☐ 2. Mararka qaarkood
☐ 3. Marna

QAYBTA III. Culayska Shaqada iyo Saacadaha Shaqada

14. Ma u aragtaa culayska shaqadaada hadda inuu badan yahay?

☐ 1. Haa
☐ 2. Maya

15. Celcelis ahaan, imisa saacadood ayaad shaqaysaa maalintii?

_____ saacadood/maalintii

16. Caadi ahaan ma shaqaysaa in ka badan 8 saacadood maalintii?

☐ 1. Haa
☐ 2. Maya

17. Immisa jeer ayaa lagaa rabaa inaad shaqayso saacadaha laguu qorsheeyay ka baxsan?

☐ 1. Marna
☐ 2. Si dhif ah
☐ 3. Mararka qaarkood
☐ 4. Badanaa
☐ 5. Had iyo jeer

18. Ma haysataa waqti kugu filan oo aad ku dhammaystirto hawlaha nadiifinta ee laguu xilsaaray?

☐ 1. Haa
☐ 2. Maya

19. Ma dareemaysaa in tirada hawlaha nadiifinta ee laguu xilsaaray ay aad u badan yihiin?

☐ 1. Haa
☐ 2. Maya

QAYBTA IV. Walbahaarka La Xiriira Shaqada

20. Intii lagu jiray bishii la soo dhaafay, ma la kulantay walbahaar la xiriira shaqadaada nadiifiyaha isbitaalka?

☐ 1. Haa
☐ 2. Maya

Koodhka:

Haa = 1
Maya = 0

21. Haddii Haa, maxay ahaayeen ilaha ugu waaweyn ee walbahaarkaaga shaqada?

Jawaabo badan waa la dooran karaa.

☐ 1. Culays shaqo oo badan
☐ 2. Saacado shaqo oo dheer
☐ 3. Shaqaale aan ku filnayn
☐ 4. Kormeer xumo
☐ 5. Shaqadayda oo aan si fiican loo aqoonsan
☐ 6. Mushahar hoose
☐ 7. Deegaan shaqo oo aan wanaagsanayn
☐ 8. PPE oo aan jirin/aan ku filnayn
☐ 9. La kulanka walxo khatar ah
☐ 10. Khilaaf lala yeesho shaqaalaha kale
☐ 11. Khilaaf lala yeesho kormeerayaasha
☐ 12. Wax kale: ____________

QAYBTA V. Calaamadaha Jirka iyo Maskaxda ee La Xiriira Walbahaarka

Tilmaamaha: Haddii aad su'aasha 20 kaga jawaabtay Haa, fadlan sheeg inta jeer ama heerka aad calaamad kasta la kulantay bishii la soo dhaafay.

No.	Calaamadda	Ma jirto	Fudud	Dhexdhexaad	Daran
22	Jirka oo ruxma/gariira	☐	☐	☐	☐
23	Dawakh	☐	☐	☐	☐
24	Dhidid badan	☐	☐	☐	☐
25	Murqo xanuun	☐	☐	☐	☐
26	Madax-xanuun	☐	☐	☐	☐
27	Dhabar-xanuun	☐	☐	☐	☐
28	Hurdo khalkhalsan	☐	☐	☐	☐
29	Neefsashada oo ku adkaata/raaxo-darro neefsashada	☐	☐	☐	☐
30	Dheefshiid xumo/raaxo-darro caloosha ah	☐	☐	☐	☐
31	Daal ama tabar-darro	☐	☐	☐	☐
32	Xanaaq	☐	☐	☐	☐
33	Feejignaanta oo ku adkaata	☐	☐	☐	☐

Koodhka la soo jeediyay:

0 = Ma jirto
1 = Fudud
2 = Dhexdhexaad
3 = Daran

QAYBTA VI. Hurdada

34. Bishii la soo dhaafay, ma la kulantay dhibaato ama khalkhal hurdo?

☐ 1. Haa
☐ 2. Maya

35. Haddii Haa, noocee dhibaato hurdo ah ayaad la kulantay?

☐ 1. Hurdo qaadashada oo kugu adkaata
☐ 2. Habeenkii oo aad marar badan soo toosto
☐ 3. Aroor hore oo aad soo toosto
☐ 4. Hurdo tayo xun
☐ 5. Wax kale: _____________

36. Celcelis ahaan, imisa saacadood ayaad seexataa habeenkii?

_____ saacadood

QAYBTA VII. Isticmaalka Khamriga

37. Hadda ma cabtaa khamri/cabitaan khamri leh?

☐ 1. Haa
☐ 2. Maya

38. 30kii maalmood ee la soo dhaafay, immisa jeer ayaad cabtay khamri?

☐ 1. Marnaba
☐ 2. In ka yar hal mar toddobaadkii
☐ 3. 1–2 maalmood toddobaadkii
☐ 4. 3–4 maalmood toddobaadkii
☐ 5. 5 maalmood ama ka badan toddobaadkii

39. Marka aad khamri cabto, ma u aragtaa cabitaankaaga inuu badan yahay?

☐ 1. Haa
☐ 2. Maya

Falanqaynta ugu weyn:

Cabitaanka khamriga badan: Haa/Maya.

QAYBTA VIII. Isticmaalka Qaadka

40. Hadda ma ruugtaa qaad (chat)?

☐ 1. Haa
☐ 2. Maya

41. 30kii maalmood ee la soo dhaafay, immisa jeer ayaad ruugtay qaad?

☐ 1. Marnaba
☐ 2. In ka yar hal mar toddobaadkii
☐ 3. 1–2 maalmood toddobaadkii
☐ 4. 3–4 maalmood toddobaadkii
☐ 5. 5 maalmood ama ka badan toddobaadkii

42. Caadi ahaan, imisa saacadood ayaad ku qaadataa ruugista qaadka maalinta aad ruugayso?

_____ saacadood

QAYBTA IX. Sigaarka

43. Hadda ma cabtaa sigaarka?

☐ 1. Haa
☐ 2. Maya

44. Hore ma si joogto ah u cabbi jirtay sigaarka?

☐ 1. Haa
☐ 2. Maya

45. Haddii aad hadda sigaarka cabto, qiyaastii imisa xabbo ayaad maalintii cabtaa?

_____ xabbo

QAYBTA X. Ku Qanacsanaanta Shaqada

46. Guud ahaan, ma ku qanacsan tahay shaqadaada hadda ee nadiifiyaha isbitaalka?

☐ 1. Haa
☐ 2. Maya

47. Intee ayaad ku qanacsan tahay mushaharkaaga?

☐ 1. Aad ayaan ugu qanacsanahay
☐ 2. Waan ku qanacsanahay
☐ 3. Dhexdhexaad
☐ 4. Kuma qanacsani
☐ 5. Aad uma qanacsani

48. Intee ayaad ku qanacsan tahay saacadaha shaqadaada?

☐ 1. Aad ayaan ugu qanacsanahay
☐ 2. Waan ku qanacsanahay
☐ 3. Dhexdhexaad
☐ 4. Kuma qanacsani
☐ 5. Aad uma qanacsani

49. Intee ayaad ku qanacsan tahay xiriirka aad la leedahay kormeerahaaga?

☐ 1. Aad ayaan ugu qanacsanahay
☐ 2. Waan ku qanacsanahay
☐ 3. Dhexdhexaad
☐ 4. Kuma qanacsani
☐ 5. Aad uma qanacsani

50. Intee ayaad ku qanacsan tahay xiriirka aad la leedahay shaqaalaha kale?

☐ 1. Aad ayaan ugu qanacsanahay
☐ 2. Waan ku qanacsanahay
☐ 3. Dhexdhexaad
☐ 4. Kuma qanacsani
☐ 5. Aad uma qanacsani

QAYBTA XI. Ku Qanacsanaanta Deegaanka Shaqada

51. Guud ahaan, ma ku qanacsan tahay deegaankaaga shaqada?

☐ 1. Haa
☐ 2. Maya

52. Ma ku qanacsan tahay nadaafadda iyo badbaadada goobtaada shaqada?

☐ 1. Haa
☐ 2. Maya

53. Ma ku qanacsan tahay helitaanka agabka iyo qalabka nadiifinta?

☐ 1. Haa
☐ 2. Maya

54. Ma ku qanacsan tahay hawo-qaadashada goobtaada shaqada?

☐ 1. Haa
☐ 2. Maya

55. Ma ku qanacsan tahay iftiinka goobtaada shaqada?

☐ 1. Haa
☐ 2. Maya

56. Ma ku qanacsan tahay helitaanka biyaha iyo adeegyada fayadhowrka?

☐ 1. Haa
☐ 2. Maya

QAYBTA XII. Aqoonsiga Shaqada iyo Taageerada Bulshada

57. Ma dareemaysaa in maamulka isbitaalku si ku filan u aqoonsan yahay shaqadaada nadiifiyaha isbitaalka?

☐ 1. Haa
☐ 2. Maya

58. Ma dareemaysaa in shaqaalaha isbitaalku ixtiraamaan shaqada aad qabato?

☐ 1. Haa
☐ 2. Maya

59. Ma heshaa mahadnaq ama bogaadin marka aad shaqadaada si wanaagsan u qabato?

☐ 1. Haa
☐ 2. Maya

60. Ma dareemaysaa in waxqabadkaaga ku aaddan badbaadada bukaanka iyo ka hortagga caabuqyada isbitaalka la aqoonsan yahay?

☐ 1. Haa
☐ 2. Maya

61. Ma la kulantaa in shaqadaada aan si ku filan bulshada ama shaqaalaha kale uga helin aqoonsi?

☐ 1. Haa
☐ 2. Maya

QAYBTA XIII. Arrimo Dheeraad ah oo Goobta Shaqada ah

62. Ma haysataa qalab nadiifin oo ku filan si aad u qabato waajibaadkaaga?

☐ 1. Haa
☐ 2. Maya

63. Ma haysataa agab/walxo nadiifin oo ku filan?

☐ 1. Haa
☐ 2. Maya

64. Ma si joogto ah u qabataa hawlo nadiifin oo jir ahaan culus?

☐ 1. Haa

☐ 2. Maya

65. Inta aad shaqaynayso ma la kulantaa ur aan fiicnayn?

☐ 1. Haa

☐ 2. Maya

66. Ma la kulantaa walxaha kiimikada ah ee loo isticmaalo nadiifinta?

☐ 1. Haa

☐ 2. Maya

67. Ma dareemaysaa badbaado marka aad gudanayso waajibaadkaaga nadiifinta?

☐ 1. Haa

☐ 2. Maya

68. Marka aad la kulanto dhibaato la xiriirta shaqada, ma ka heshaa taageero ku filan kormeerahaaga tooska ah?

☐ 1. Had iyo jeer

☐ 2. Mararka qaarkood

☐ 3. Marna

Aad baad ugu mahadsan tahay ka-qaybgalkaaga daraasaddan.
