# Supplementary material for: Work‐Related Stress Among Neglected Work Forces in Tertiary Hospitals, Eastern Ethiopia: A Cross‐Sectional Study
Source: Health Sci Rep. 2026 Oct 10;9(10):e73374. doi: 10.1002/hsr2.73374 (PMC13653264; doi:10.1002/hsr2.73374)
Supplement: Supplementary file 2 [file HSR2-9-e73374-s001.docx]

**Supplementary Figures**

**Figure S1**: Presented the general framework (or conceptual skeleton) of the current study on work-related stress (WRS) among tertiary hospital cleaners, Eastern Ethiopia

**Figure S2**: Represented the key message of the study WRS among neglected work Forces (Cleaners) in tertiary hospitals using hospital based on cross-sectional study, Eastern Ethiopia
